# The toothless pterosaur *Jidapterus edentus* (Pterodactyloidea: Azhdarchoidea) from the Early Cretaceous Jehol Biota and its paleoecological implications

**DOI:** 10.1371/journal.pone.0185486

**Published:** 2017-09-26

**Authors:** Wen-Hao Wu, Chang-Fu Zhou, Brian Andres

**Affiliations:** 1 Key Laboratory for Evolution of Past Life and Environment in Northeast Asia, Ministry of Education, Jilin University, Changchun, Jilin, China; 2 Research Center of Palaeontology & Stratigraphy, Jilin University, Changchun, Jilin, China; 3 College of Earth Science and Engineering, Shandong University of Science and Technology, Qingdao, Shandong, China; 4 Paleontological Institute, Shenyang Normal University, Shenyang, Liaoning, China; 5 Department and Earth and Environmental Science, University of Pennsylvania, Philadelphia, Pennsylvania, United States of America; University of Michigan, UNITED STATES

## Abstract

**Background:**

In the Early Cretaceous Jehol Biota, the toothless pterosaurs flourished with the chaoyangopterids and tapejarids playing a key role in understanding the early diversity and evolution of the Azhdarchoidea. Unlike the more diverse tapejarids, the rarer chaoyangopterids are characterized by a long and low rostrum, supporting a close relationship with the huge azhdarchids. Unfortunately, our knowledge is still limited in the osteology, paleoecology, and taxonomy of the Chaoyangopteridae. As one of the best preserved skeletons, the type and only specimen of *Jidapterus edentus* provides an opportunity to understand the morphology and paleoecology of the chaoyangopterids.

**Results:**

Our study of the osteology of *Jidapterus edentus* reveals valuable information about the morphology of the Chaoyangopteridae such as a rostrum with a curved dorsal profile, high Rostral Index (RI), larger angle between the dorsal and postorbital processes of the jugal, sequentially shorter fourth to seventh cervical vertebrae, sternum with a plate wider than long, contact of the metacarpal I with the distal syncarpal, pneumatic foramen on first wing phalanx, hatchet-like postacetabular process with unconstricted neck and small dorsal process, distinctly concave anterior margin of pubis, subrectangular pubic plate with nearly parallel anterior and posterior margins, longer proximal phalanges of pedal digits III and IV, as well as reduced and less curved pedal unguals. These features further support the validity of *Jidapterus edentus* as a distinct species and the close relationship of the chaoyangopterids with the azhdarchids. Paleoecologically, the chaoyangopterids are probably like the azhdarchids, more terrestrial than the contemporaneous and putatively arboreal tapejarids, which may have been limited to the forest-dominated ecosystem of the Jehol Biota.

**Discussion:**

The osteology of *Jidapterus edentus* further supports the close relationship of the Chaoyangopteridae with the Azhdarchidae in sharing a high RI value and reduced and mildly-curved pedal unguals, and it also implies a possible paleoecological similarity in their terrestrial capability. Combined with the putatively arboreal and herbivorous tapejarids, this distinct lifestyle of the chaoyangopterids provides new insights into the diversity of pterosaurs in the ecosystem of the Jehol Biota.

## Introduction

As one of the most significant Cretaceous pterosaur groups, the toothless Azhdarchoidea is famous for its huge-sized azhdarchids and high-crested tapejarids [1–3]. The Azhdarchidae Nessov 1984 [4] is one of the last pterosaur lineages, living up to the Cretaceous–Paleogene boundary. Azhdarchids are poorly known in deeper time, and their origins remain unclear [2, 3]. In the last decade, the Chaoyangopteridae [5], which share similar cranial structures with the Azhdarchidae, became well known in the Early Cretaceous Jehol Biota, shedding light on the early radiation of Azhdarchoidea [5–8].

The Jehol Biota includes five species previously assigned to Chaoyangopteridae: *Chaoyangopterus zhangi* Wang and Zhou 2003 [9], *Jidapterus edentus* Dong et al. 2003 [10], *Eopteranodon lii* Lü and Zhang 2005 [11], *Eoazhdarcho liaoxiensis* Lü and Ji 2005 [12] and *Shenzhoupterus chaoyangensis* Lü et al. 2008 [5]. However, their taxonomical validity and classification are contentious [5, 12–15]. For example, Wang and Zhou [14] argued that *Chaoyangopterus* was the only valid taxon, with *Jidapterus*, *Eopteranodon*, and *Eoazhdarcho* as its junior synonyms, and referred it to the Pteranodontidae. Later, the suggestion was made and widely accepted that these taxa belong to the Azhdarchoidea [5–8, 13, 15–17]. Due to lack of anatomical information in the original descriptions of these taxa, their validity remains unresolved [3, 6, 18, 19]. This seems to be especially true for *Jidapterus*, which is based on the best preserved skeleton, but has been excluded from some phylogenetic analyses [7, 16]. We redescribe the holotype and only specimen of *Jidapterus edentus* here to resolve the taxonomic validity of *Jidapterus* and reveal more information about the morphology and paleoecology of the Chaoyangopteridae.

One of the operative questions of this, and every paleontological description, is whether the material described belongs to the same species as its sister taxon. It is a consequence of evolution that closely related species appear similar, and incomplete preservation in paleontological specimens, especially non-overlapping preservation, can obscure some of their differences. The traditional diagnosis and comparative description of specimens is both sufficient and necessary to distinguish species, but this is taken to another step here by phylogenetic analysis of *Jidapterus*. Whereas, phylogenetic analysis cannot corroborate a hypothesis that sister taxa belong to the same species because it assumes bifurcate evolution and sister taxa can also be sister species, it can falsify such a hypothesis. If a species is recovered as a sister taxon to a large clade, it is not supported as belonging to one of the species of that clade. In addition, if another taxon is recovered as more closely related to the possible members of the same species, then those original members are not supported as conspecifics. *Jidapterus* has been recovered as the sister group to *Chaoyangopterus* in every phylogenetic analysis of their relationships, and even suggested to be the same species by Witton [3]. To test the validity of *Jidapterus edentus* as a distinct species and determine its evolutionary relationships, a phylogenetic analysis of all putative chaoyangopterids and most pterosaur species was conducted. These putative chaoyangopterids include *Jidapterus edentus* (RCPS-030366CY), *Chaoyangopterus zhangi* (IVPP V13397 and PMOL-AR00076), *Shenzhoupterus chaoyangensis* (HGM 41HIII-305A), *Eoazhdarcho liaoxiensis* (GMN-03-11-002), *Eopteranodon lii* (BPV-078), *Lacusovagus magnificens* Witton 2008 [20] (SMNK PAL 4325), and two undescribed specimens in the collections of the Liaoning Paleontological Museum of Chaoyang National Geopark (LPM L112113 and N081607; S1 and S2 Figs).

## Materials and methods

The holotype of *Jidapterus edentus* (RCPS-030366CY) is a nearly complete and articulated skeleton with skull, mandible, and postcranium preserved. The rostral portion of skull is well preserved, whereas the other skull remains are scattered. The fore- and hindlimbs as well as the mid-cervicals are articulated; whereas the rest of the postcranial skeleton (e.g. dorsal and sacral vertebrae as well as pectoral and pelvic girdles) is disarticulated and scattered. The specimen was prepared by an unnamed technician before this study and deposited in the Research Center of Palaeontology & Stratigraphy of Jilin University, Changchun, Jilin Province, China (RCPS).

The phylogenetic analysis of *Jidapterus edentus* and the relationships of the Pterosauria was executed using TNT version 1.5 [21]. It is based on the analysis of Zhou et al. [22], which is turn based on the previous analyses of Chinese pterosaurs by Andres and Ji [15] and Andres et al. [8, 23]. It has been updated with the taxonomy of Bennett [24–26], Lü et al. [5], Martin-Silverstone et al. [27], and Pinheiro and Rodrigues [28], as well as the addition several pterosaur species. This analysis incorporates all the characters and most of the species considered currently valid for pterosaurs, producing a matrix of 271 characters and 134 species. Five non-pterosaur outgroups were coded, but *Euparkeria capensis* Broom 1913 [29] was used as the main analytical outgroup and so is listed first in the matrix. Characters were divided into continuous and discrete partitions, with the continuous characters rescaled to unity using the “nstates stand” command [30]. Ordered and unordered characters were used, and all characters were equally weighted. Polymorphic and reductive codings (sensu Strong and Lipscomb [31]) were also implemented. Ambiguous branch support was not used, zero-length branches were automatically collapsed, and resultant trees were filtered for best score. Basic tree-searches of 2000 random addition sequence replicates were conducted with all trees kept. The phylogenetic matrix, characters and states, and analytical settings are provided as a TNT executable supplementary file (S1 Appendix) that will repeat the analysis when loaded with the “procedure” command.

### Institutional abbreviations

BPV, Beijing Museum of Natural History, Beijing; BXGM, Benxi Geological Museum, Benxi; RCPS, Research Center of Palaeontology & Stratigraphy, Jilin University, Changchun; D, Dalian Natural History Museum, Dalian; GMN, Geological Museum of Nanjing, Nanjing; HGM, Henan Geological Museum, Zhengzhou; IMCF, Iwaki Coal and Fossil Museum, Fukushima; IVPP, Institute of Vertebrate Paleontology and Paleoanthropology, Beijing; LPM, Liaoning Paleontological Museum of Chaoyang National Geopark, Chaoyang; PMOL, Paleontological Museum of Liaoning, Shenyang Normal University, Shenyang; SDUST, Collections of Vertebrate Paleontology, Shandong University of Science and Technology; SMNK, Staatliches Museum für Naturkunde Stuttgart, Stuttgart; TMM, Texas Memorial Museum, University of Texas at Austin, Austin, TX; ZMNH, Zhejiang Museum of Natural History, Hangzhou.

## Results

### Systematic paleontology

Pterosauria Kaup 1834 [32]

Pterodactyloidea Plieninger 1901 [33]

Azhdarchoidea Unwin 1995 [34]

Chaoyangopteridae Lü et al. 2008 [5]

*Jidapterus edentus* Dong, Sun & Wu 2003 [10]

#### Holotype

**Fig 1:**
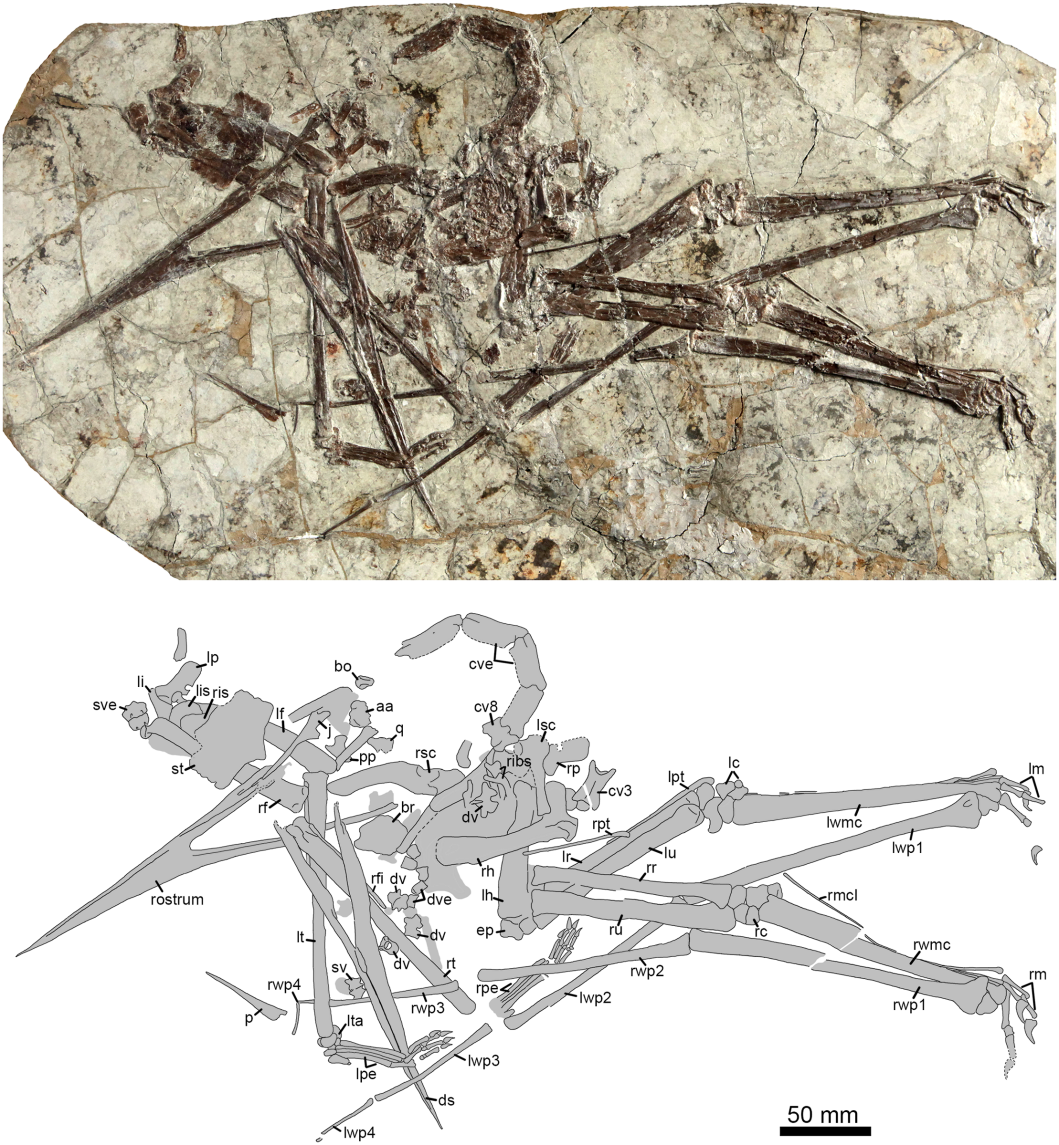
Entire skeleton of *Jidapterus edentus* (RCPS-030366CY) preserved in a slab mostly in right lateral view. Abbreviations: aa, the atlas-axis complex; bo, basioccipital; br, braincase; cve, cervical vertebrae; cv3, the third cervical vertebra; cv8, the eighth cervical vertebra; ds, dentaries; dv, dorsal vertebra; dve, dorsal vertebrae; ep, epiphysis; j, jugal; lc, left carpus; lf, left femur; li, left ilium; lis, left ischium; lm, left manual digits; lp, left pubis; lpe, left pes; lpt, left pteroid; lr, left radius; lsc, left scapula-coracoid; lt, left tibia; lta, left tarsus; lu, left ulna; lwmc, left wing metacarpal; lwp1–4; left wing phalanges 1–4; p, pterygoid; pp, postacetabular process of the right ilium; q, quadrate; rc, right carpus; rh, right humerus; rf, right femur; rfi, right fibula; ribs, ribs; ris, right ischium; rm, right manual digits; rmcI, right metacarpal I; rp, right pubis; rpe, right pes; rpt, right pteroid; rr, right radius; rsc, right scapula-coracoid; rt, right tibia; ru, right ulna; rwmc, right wing metacarpal; rwp1–4, right wing phalanges 1–4; rostrum, rostrum; st, sternum; sv, sacral vertebra; sve, sacral vertebrae. Gray shaded areas bound by solid lines indicate preserved bones, dashed lines indicate bones with margins discerned by impression, and lack of lines indicate missing bone or displacement along cracks in the slab.

**Fig 2:**
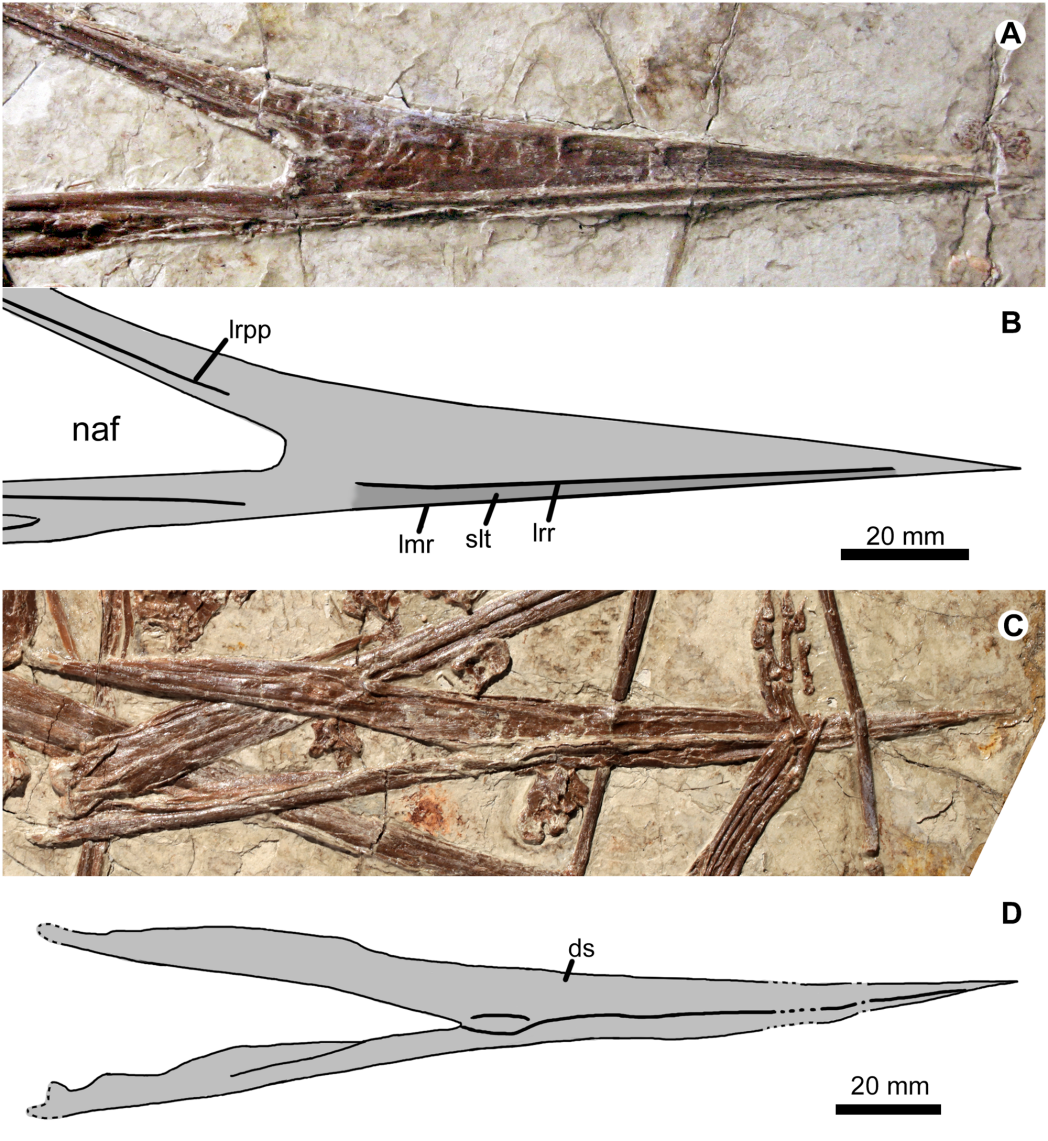
Rostrum in right lateral view (A, B) and lower jaws mostly in ventral view (C, D) of *Jidapterus edentus* (RCPS-030366CY). Abbreviations: ds, dentaries; lmr, labial margin of the rostrum; lrpp, a low ridge of the premaxillary process; lrr, a low ridge of rostrum; naf, nasoantorbital fenestra; slt, shallow longitudinal trough. Gray shaded areas bound by solid lines indicate preserved bones and the dark gray area indicates shallow longitudinal trough (slt).

**Fig 3:**
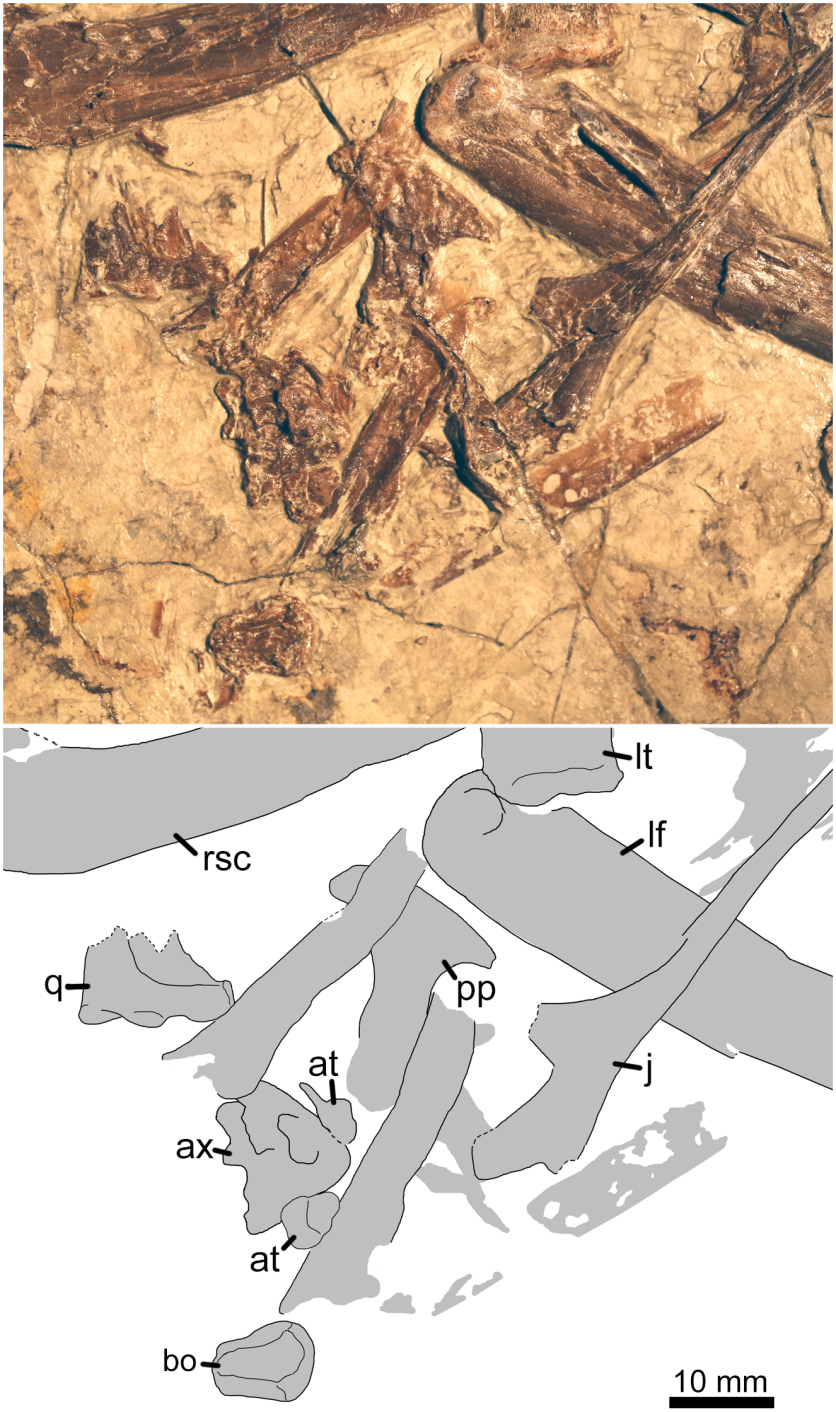
Disarticulated cranial elements, atlas-axis complex, and postcranial elements of *Jidapterus edentus* (RCPS-030366CY). Abbreviations: at, atlas; ax, axis; bo, basioccipital; j, jugal; lf, left femur; lt, left tibia; pp, postacetabular process of the right ilium; q, quadrate; rsc, right scapula. Gray shaded areas bound by solid lines indicate preserved bones, and dashed lines or lack of lines indicate missing bone.

**Fig 4:**
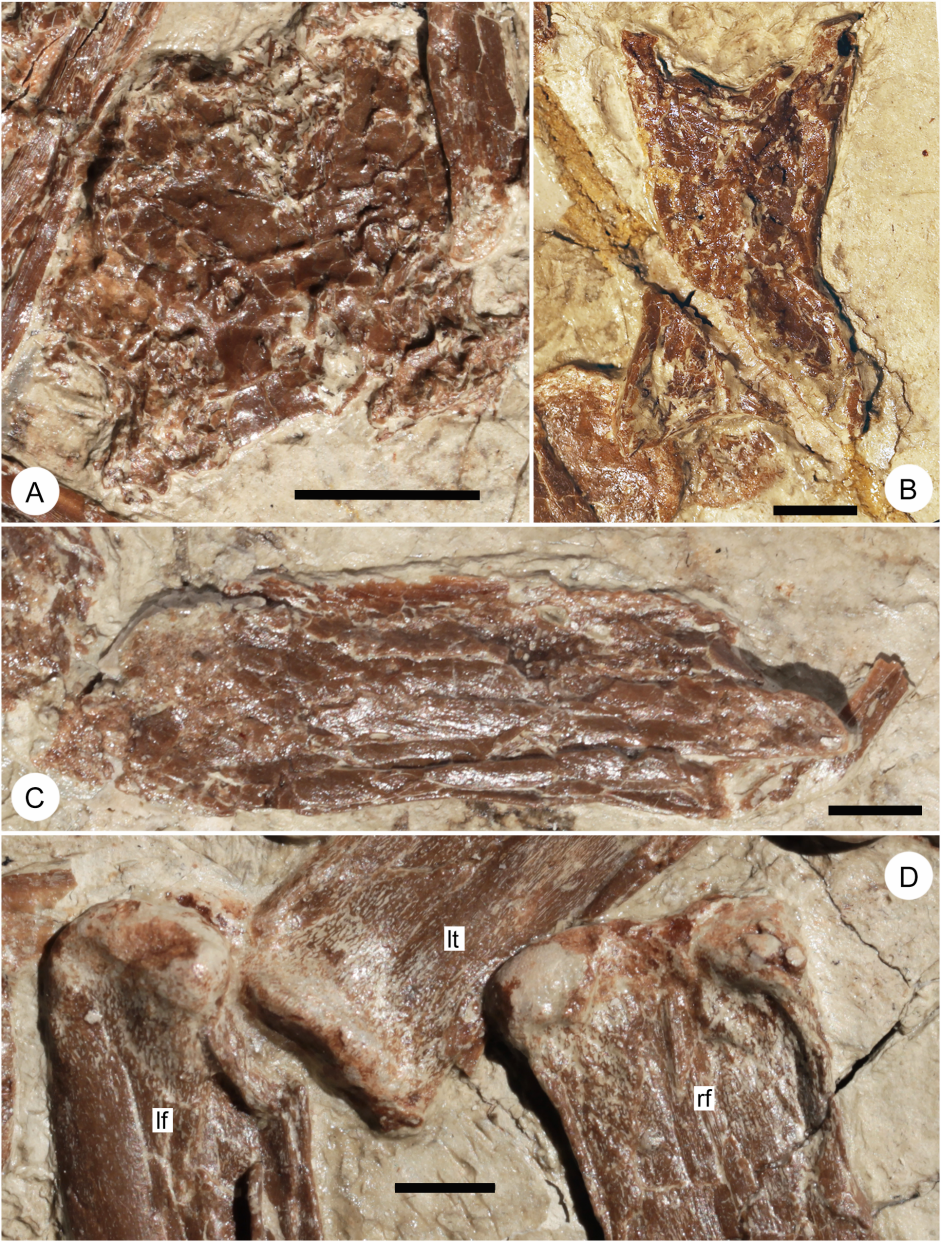
Enlarged images of endocast (A), third cervical vertebra in dorsal view (B), fourth cervical vertebra in right lateral view (C), and distal ends of femora in posterior view (D) of *Jidapterus edentus* (RCPS-030366CY). Abbreviations: lf, distal portion of the left femur; lt, proximal portion of the left tibia; rf, distal portion of the right femur. Scale bar = 10 mm.

**Fig 5:**
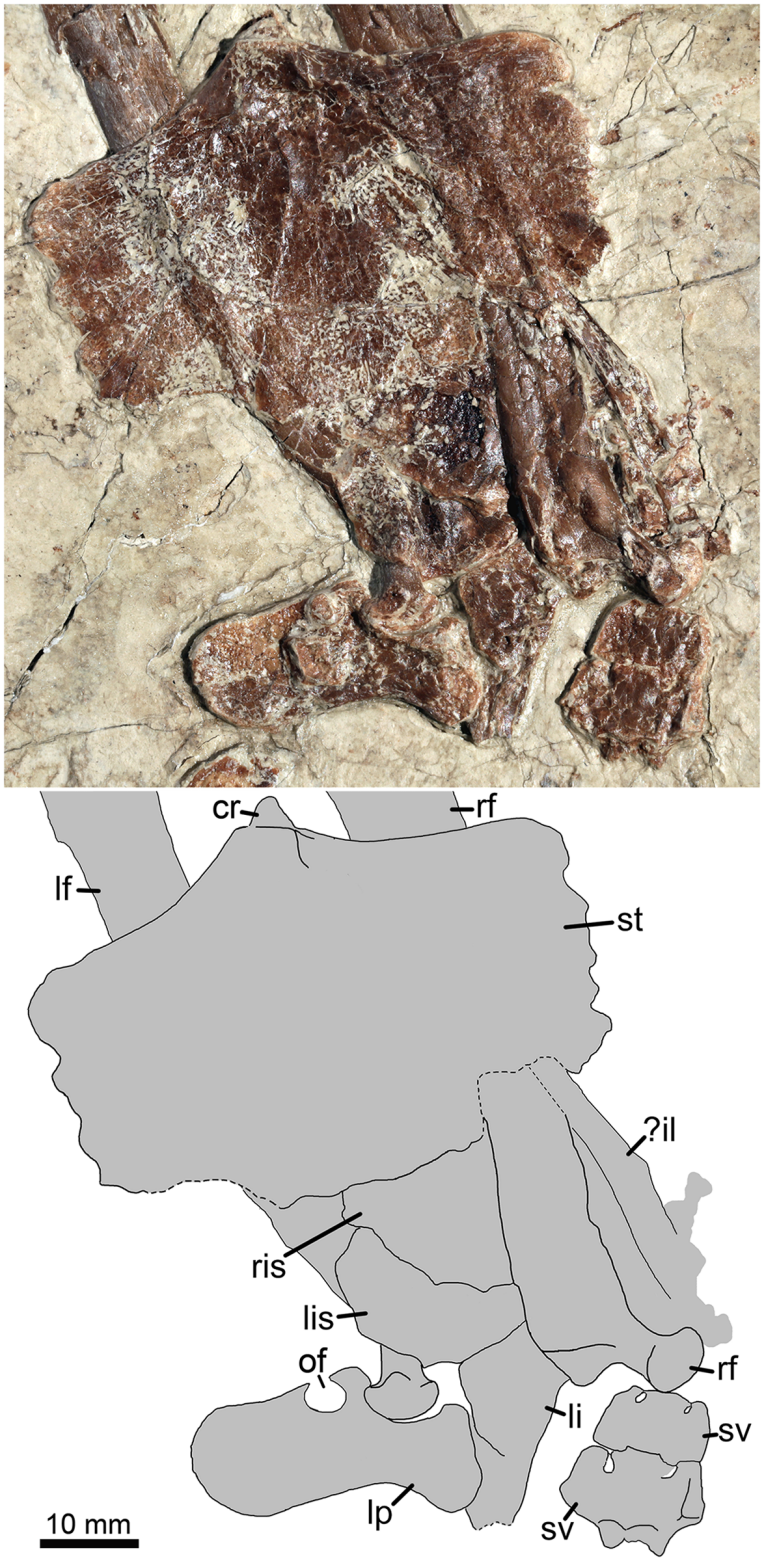
Sacral vertebrae and sternum in ventral view as well as the disarticulated pelvic girdle in medial view of *Jidapterus edentus* (RCPS-030366CY). Abbreviations: cr, cristospine; lf, left femur; li, left ilium; lis, left ischium; lp, left pubis; of, obturator foramen; rf, right femur; ris, right ischium; st, sternum; sv, sacral vertebra;? il, possible ilium fragment. Gray shaded areas bound by solid lines indicate preserved bones, dashed lines indicate bones with margins discerned by impression, and lack of lines indicate missing bone.

**Fig 6:**
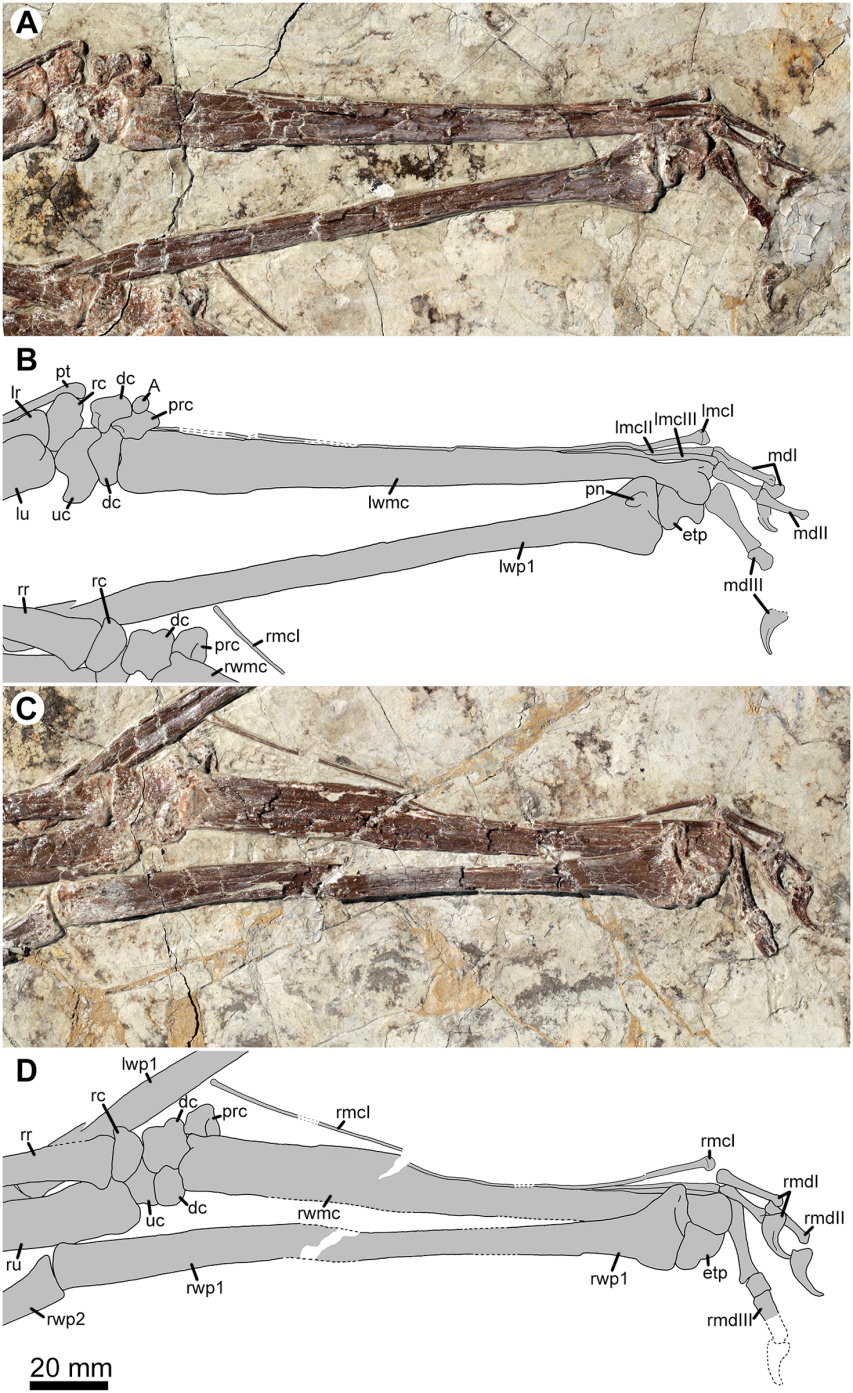
Left manus in ventral view (A) and right manus in dorsal view (B) of *Jidapterus edentus* (RCPS-030366CY). Abbreviations: A, sesamoid A; dc, distal carpals; etp, extensor process; lmcI–III, left metacarpals I–III; lr, left radius; lu, left ulna; lwmc, left wing metacarpal; lwp1, the left first wing phalanx; mdI–III, manual digits I–III; pn, pneumatic foramen; prc, preaxial carpal; pt, pteroid; rc, radial carpal; rmcI, right metacarpal I; rmdI–III, right manual digits I–III; rr, right radius; ru, right ulna; rwmc, right wing metacarpal; rwp1–2, right wing phalanx 1 and 2; uc, ulnar carpal. Gray shaded areas bound by solid lines indicate preserved bones, dashed lines indicate bones with margins discerned by impression, and lack of lines indicate missing bone or displacement along cracks in the slab.

**Fig 7:**
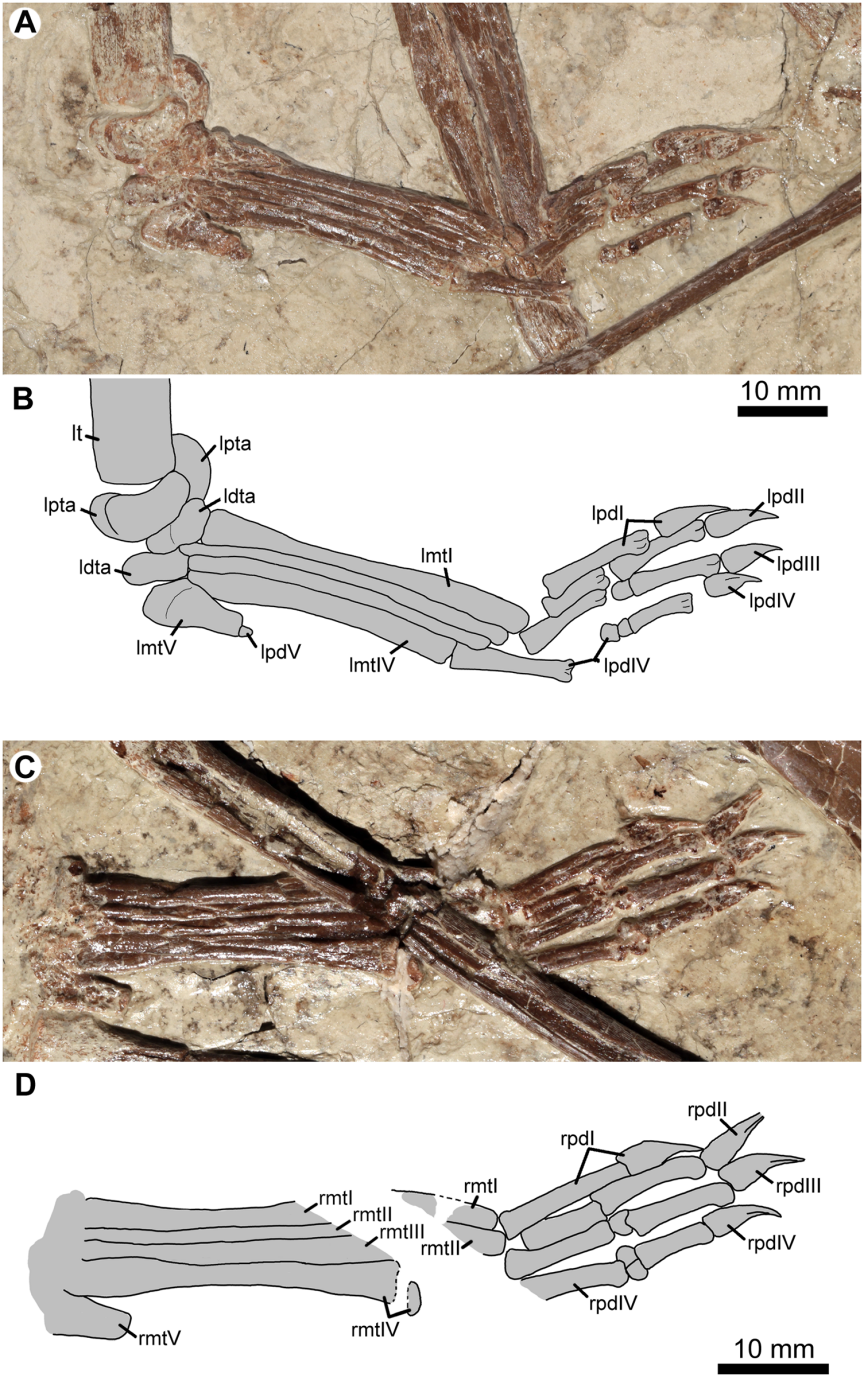
Left pes in ventral view (A, B) and right pes in dorsal view (C, D) of *Jidapterus edentus* (RCPS-030366CY). Abbreviations: ldta, left distal tarsal; lmtI–V, left metatarsals I–V; lpdI–V, left pedal digits I–V; lpta, left proximal tarsal; lt, left tibia; rmtI–V, right metatarsals I–V; rpdI–IV, right pedal digits I–IV. Gray shaded areas bound by solid lines indicate preserved bones, dashed lines indicate bones with margins discerned by impression, and lack of lines indicate missing bone or obscuring by overlying bones.

RCPS-030366CY (= CAD-01), a nearly complete and mostly articulated skeleton including skull, mandible, and postcranial remains (Figs 1–7).

#### Locality and horizon

Chaoyang City, western Liaoning Province, China. Early Cretaceous Jiufotang Formation (Aptian, 122.1Ma [35]).

#### Revised diagnosis

Chaoyangopterid pterosaur species that is diagnosed by the following combination of characters: long and low rostrum (Rostral Index: 5.1) with straight ventral margin and curved dorsal margin, longitudinal ridge along labial margin of the rostrum, long and slender premaxillary process, long symphysis over half of mandible length; moderately elongate mid-cervical vertebrae, absence of pneumatic foramen on lateral side of mid-cervicals; sternal plate wider than long, pneumatic foramen on first wing phalanx, hatchet-like postacetabular process with unconstricted neck and small dorsal process, a distinctly concave anterior margin of pubis, pubic plate sub-rectangular with nearly parallel anterior and posterior sides; and pedal digit III-1 shorter than pedal digit IV-1, longer proximal phalanges of pedal digits III and IV, and reduced and less curved unguals. It differs from *Chaoyangopterus* in having a longitudinal ridge along labial margin of the rostrum, a larger angle between the dorsal and postorbital processes of the jugal, and sequentially shorter fourth to seventh cervical vertebrae. It differs from *Shenzhoupterus* in having a high Rostral Index (5.1), straight occlusal margin of the rostral jaws, and a longitudinal ridge along labial margin of the rostrum, sequentially decreased fourth to seventh cervical vertebra, and different limb bone proportions.

### Description

As in the other chaoyangopterid specimens, the specimen represents an immature individual, inferred from the following ontogenetic characters: complete disarticulation of the skull, neural arch and centrum unfused in dorsal vertebrae, sacrum unfused and not fused to pelvis, scapula and coracoids unfused, epiphysis present and not fused with the distal end of the humerus, syncarpals unfused, extensor tendon process not fused with the first wing phalanx, tibiotarsus unfused, and fibrous bone texture in limb bones [36, 37]. Although not as osteologically mature as the *Chaoyangopterus* specimens, both considered sub-adults, it is roughly the same size with a wingspan estimate of 1.53 m (calculated by adding the lengths of the humerus, ulna/radius, wing metacarpal, and four wing phalanges on both sides [37]). The holotype and referred specimen of *Chaoyangopterus* have wingspan estimates of 1.71 and 1.45 m, respectively [9, 19]. The holotype of *Jidapterus edentus* is therefore considered to be a juvenile individual.

#### Skull and mandible

The rostrum is well preserved (Figs 1 and 2) comprising the premaxilla and maxilla, the suture of which is not visible. It is low, tapering, and elongate with a length of 115.7 mm. This rostrum is similar to that of azhdarchids and chaoyangopterids and much longer than the abbreviated rostra of tapejarids and thalassodromines. The ventral margin of the rostrum is straight as in thalassodromines and azhdarchids, but different from tapejarids that show an anteriorly downturned condition. However, in *Shenzhoupterus*, the ventral margin of the rostrum is slightly concave, associated with a convex condition of the lower jaw [5]. A low ridge extends longitudinally and forms the labial margin of the rostrum. Along the labial margin is a shallow longitudinal trough that is dorsally bordered by another low ridge, suggesting that a keratinous sheath was present in life. This structure is unknown in other chaoyangopterids, and possibly diagnostic for *Jidapterus*. The rostrum has a smooth and straight dorsal profile that becomes deflected dorsally at its preserved posterior fifth portion. The curvature is highlighted posteriorly by the upturned premaxillary process above the nasoantorbital fenestra. Among azhdarchoids, this dorsal concavity is characteristic for chaoyangopterids [5, 9, 19]. The prenarial rostrum appears to have a straight dorsal margin as in other azhdarchoids, except for tapejarids whose rostrum is dorsally accentuated by a premaxillary crest.

The rostrum is 22.6 mm high at the anterior margin of the nasoantorbital fenestra. The rostral index (RI), the ratio of the prenarial rostrum length to the height at the anterior margin of the nasoantorbital fenestra [38], is 5.1. This is significantly larger than 3.7 in *Shenzhoupterus* [5], but within the range of 4.8–5.3 in *Chaoyangopterus* (4.8 in IVPP V13397, estimated from Fig 2f in Wang and Zhou [14]; 5.3 in PMOL-AR00076). The premaxillary process is well preserved forming the dorsal rim of the nasoantorbital fenestra, but its posterior end is damaged and isolated from other elements. This process is slender, elongate, and gradually tapering posteriorly, with a length of 122 mm along the nasoantorbital fenestra. The premaxillary process is slightly longer than the ventral margin of the rostrum, different from the relatively shorter condition in the azhdarchid *Zhejiangopterus* [39, 40]. The process is nearly straight and angled with the ventral margin about 27 degrees.

A low ridge extends anteroposteriorly along the lateral surface of the premaxillary process (Figs 1 and 2). Anteriorly, it is rounded and positioned close to the ventral margin of the premaxillary process. Distally, the ridge becomes confluent with the inner margin of the premaxillary process, forming the dorsal margin of the nasoantorbital fenestra. The nasoantorbital fenestra appears to be large, although its posterior border is not preserved. In contrast, its anterior corner is clearly bounded by the premaxilla-maxilla complex. Ventral to this fenestra, the maxilla is incompletely preserved with a damaged posterior end. It is slender with an estimated length of 77 mm. The palatal aspect of the maxilla is partially exposed, and separated posteriorly from the main part of the maxilla by a longitudinal trough, possibly representing the palatine fenestra.

The jugal is displaced from its original position (Figs 1 and 3). It is triradiate with a long and slender anterior process, broader lacrimal process, and dorsoposteriorly inclined postorbital process. Below the postorbital process of the jugal, the posterior end of the jugal is damaged along the ventral margin and may include the contact with the quadratojugal. The anterior process points anteriorly; posteriorly, the process is thickened and confluent with the dorsal process. The lateral surface of the anterior process is rugose. The dorsal process is possibly broken off at its dorsal tip. The rest of it is a broad and short base with the posterior margin nearly vertical with respect to the anterior process, and forms a right angle with the postorbital process. In *Chaoyangopterus* (PMOL-AR00076) [19], it is much more acute with a 60 degrees angle. The postorbital process is much broader and shorter than the anterior process. The postorbital process is strongly dorsoposteriorly inclined and nearly parallel to the anterior process. Between the dorsal and postorbital processes, the jugal is constricted dorsoventrally, differing from the deep condition in other azhdarchoids. Ventrally, the main body of the jugal is slightly curved ventrally and gradually deeper posteriorly.

Near the right fourth wing phalanx, a lamina-like element is identified as a pterygoid (Fig 1). It is broad laterally, sharply pointed anteriorly, and thickened along the putative medial edge. This medial edge is nearly straight and more ventrally extended than the lateral edge. The identified pterygoid is 56 mm in length.

A fragmentary quadrate (Fig 3) is preserved near the right scapula. It preserves the ventral portion containing the craniomandibular joint and the pterygoid process. Along the ventral margin, the quadrate is about 14.2 mm in width with its lateral half occupied by the craniomandibular joint. The craniomandibular joint is helical and slightly extended more medially than laterally. Medially, the pterygoid process is lamina-like, and positioned more dorsally relative to the craniomandibular joint. The pterygoid process is poorly ossified at its articular facet for the pterygoid.

A possible basioccipital (Fig 3) is located between the atlas-axis complex and the fourth mid-cervical vertebra. It is exposed in anterior view and is 10 mm long by 7.7 mm wide. The basioccipital is outlined by the convex dorsal and ventral margins, and two relatively straight lateral margins. The ventral margin is narrow, about half the width of the dorsal margin. Dorsally, the basioccipital is expanded bilaterally to form a rounded dorsal margin, where the occipital condyle is likely obscured. On the anterior surface, the basioccipital bears a smooth and longitudinal oriented depression, forming the ventral floor of the braincase. Laterally, the basioccipital is sculptured by slightly depressed and rugose troughs that are likely the articular facets for the exoccipital.

A large and plate-like element is identified as a part of the braincase (Figs 1 and 4A) delimiting a large and rounded cerebral region. It is sculptured by many shallow and strongly curved ridges, especially on its posterolateral corners. Similar structures occur in *Chaoyangopterus* and *Tapejara* but appear to be relatively smoother [19, 41].

The mandible is exposed mostly in ventral view (Fig 2C and 2D). As the largest elements of the mandible, the dentaries are preserved in both sides, whereas the other mandibular elements are not identifiable. As in *Chaoyangopterus*, the mandible is low and anteriorly pointed with an elongated symphysis. This symphysis is 110.7 mm long, about 53.8% of the mandible length. Along the symphysis is a low ventral keel, but this is possibly a taphonomic artifact. At the level of the posterior end of the symphysis, the mandible reaches the maximum depth. Posterior to the symphysis, the dentary reduces in depth. The lateral surface of the mandible is smooth without visible foramina.

#### Axial skeleton

The cervical series is nearly complete in preservation. A possible atlas-axis complex is preserved but isolated from the rest of the cervical series (Fig 3). Another isolated cervical is preserved near the right humerus, possibly representing the third cervical. The last four mid-cervicals and one posterior cervical are preserved in articulation, representing the cervicals four to eight. The ninth cervical is missing.

The identified atlas-axis complex is unfused. The atlas consists of three disarticulated elements. A tiny and rounded element is articulated with the axis with a curved suture, probably representing the intercentrum and centrum of the atlas. This intercentrum is rounded and convex on its exposed face. The centrum is poorly ossified on its exposed face, concave anteriorly, and convex posteriorly. It is about 4.9 mm wide and 2.2 mm long. A tiny element is also identified as the neural arch of the atlas. This neural arch has a broad plate and a neural spine. Ventrally, the arch has a rugose edge that is possibly the articular facet for the intercentrum/centrum. Its neural spine is flattened. The axis is crushed and cannot be described in much detail. It is triangular in outline with a tall neural spine. Both the right postzygapophysis and postexapophysis are visible.

The third cervical is exposed in dorsal view (Fig 4B). It is moderately elongate, about 29.8 mm in maximum length and 9.2 mm in minimum width. The ratio of the length versus the width is 3.2, comparable to that of non-azhdarchids (3–4) [19, 42, 43]. Typical for pterosaurs, this cervical is procoelous, a cotyle concave anteriorly and a condyle convex posteriorly. The condyle is well exposed in dorsal view. It is rounded and extends posteriorly well beyond the neural arch. Anteriorly, the cotyle can be identified under the curved margin between the prezygapophyses. As in other pterosaurs, the neural arch is expanded laterally at the prezygapophyses and postzygapophyses. Between these zygapophyses, the neural arch is constricted in width. Of these, the anterior margin is more curved than the posterior margin. The prezygapophyses are horn-shaped and at a low angle with the longitudinal axis of the vertebra. In contrast, the postzygapophyses are at a higher angle to the vertebral axis. The neural spine is damaged along the midline.

Cervicals four to eight are articulated and exposed in lateral view (Fig 1). The mid-cervicals of this series are longer than the third cervical, such as the fourth (43.2 mm), the fifth (38.2 mm), the sixth (33.7 mm) and the seventh (32.3 mm) cervicals. There is a sequential decrease in length from the fourth to seventh cervical vertebra, so that the seventh cervical is 75% of the length of the fourth cervical. However in *Chaoyangopterus* (IVPP V13397, PMOL-AR00076) and *Shenzhoupterus* (HGM 41HIII-305A), the fourth to seventh cervical vertebrae are almost in the same length [5, 9, 19]. *Jidapterus* is unusual in having its fourth cervical as its longest vertebra, suggesting that some isolated elongate cervicals identified as fifth cervicals might be fourth cervicals. The neural spines are all damaged, so their heights are uncertain. A tiny foramen is apparently present in the lateral surface of the fourth cervical (Figs 1 and 4C), which likely represents a nutrient foramen rather than a pneumatic foramen that is larger in tapejarids and thalassodromines [41, 44]. Other azhdarchoids are characterized by a pair of pneumatic foramina lateral to the neural canal that pierce the anterior posterior facets of the neural arch, but these aspects are obscured in this specimen and so their presence remains uncertain. The prezygapophyses are more ventrally positioned relative to the postzygapophyses. The prezygapophyses extend anterior the centrum. The postzygapophyses are relatively broad in lateral view and separated from the condyle by a deep ventral notch. The centrum is slightly concave along the ventral margin. The posterior condyle is slightly upturned posteriorly. The fifth and sixth cervicals are damaged. The seventh cervical is the last mid-cervical vertebra in pterosaurs. Its prezygapophyses are relatively reduced anterior to the centrum, although its postzygapophyses are still positioned high relative to the prezygapophyses. Along the ventral margin, there is a small notch anterior to the posterior condyle, which is possibly a taphonomic artifact.

The eighth cervical is the first of the two posterior cervicals found in pterosaurs. It measures 18.5 mm anteroposteriorly in length, much shorter than the mid-cervicals. The centrum is exposed with the anterior cotyle and posterior condyle visible. The ventral margin of the centrum is concave. Dorsally, a longitudinal depression is present along the base of the neural arch. The prezygapophyses are positioned ventrally and extend beyond the centrum. The postzygapophyses extend slightly beyond the condyle. A tubercle is developed on the postzygapophyses. The neural spine is well developed, but its height is uncertain because its dorsal tip is broken.

The dorsal vertebrae are disarticulated but clustered together in the specimen. In most of the preserved dorsals, the neural arch and centrum are unfused. At least ten dorsals can be identified, all comparable in size with an average length of 9 mm and width of 7.4 mm. The dorsal centra are smooth in texture, slightly constricted in the middle, and procoelous. Dorsally, a longitudinal groove forms the ventral rim of the neural canal. One dorsal vertebra is exposed in anterior view, located between the mandible and the right tibia (Fig 1). Its centrum is round and slightly depressed centrally, forming a crescentic anterior cotyle. Dorsally, the centrum is conjoined with the neural arch and encloses the neural canal. This neural canal is large in size with a diameter of about 4.5 mm. The minimum width of the dorsal vertebra is at this contact. More dorsally, the neural arch is slightly expanded bilaterally. Up to the prezygapophyses, the neural arch is as wide as the centrum.

A neural arch of a dorsal vertebra is well preserved in anterodorsal view and located among the ribs (Fig 1). It is about 16 mm high, and 21.4 mm wide across the transverse processes. The thin neural spine forms about half height of the neural arch. The transverse processes extend laterally to support the diapophyses. A distinct ridge develops from the ventral margin of the neural arch to the ventral side of the transverse process. Anteroventral to the neural spine, prezygapophyses are confluent medially. Another dorsal vertebra next to the right tibia shows the left lateral surface of the neural arch (Fig 1), but the neural spine and transverse process are broken. The neural spine points dorsally and measures about 4.5 mm at its base. The prezygapophyses extend slightly beyond the centrum anteriorly. They are larger than the postzygapophyses and positioned more dorsally than the latter. The postzygapophyses are small and positioned near the posterior margin of the centrum. The transverse processes are located more ventrally than the zygapophyses. Below the level of the transverse processes, the neural arch has pneumatic foramina and constricted anteroposteriorly. The constriction is stronger posteriorly than anteriorly forming a deep notch under the postzygapophyses. Therefore, the contact of the neural arch and centrum is much shorter than the centrum.

Seven ribs are identifiable, packed tightly and overlapped with each other. A possible posterior cervical rib is present with a characteristic robust capitulum and tuberculum. The tuberculum is stout, and the capitulum is more slender with a long neck well separated from the tuberculum. In the dorsal ribs, the tuberculum is reduced and confluent with shaft. In contrast, the capitulum is strongly reduced in size with a slender neck.

The sacrals are not fused in the sacrum. Three sacrals can be identified. Two are articulated and located next to the right femoral head (Figs 1 and 5). They are exposed in ventral view and have a smooth surface. Their shape is subtrapezoidal with a broad anterior margin that gradually tapers to a narrower posterior margin, but the anterior sacral is subequal with the posterior one in length. In the anterior sacral, the transverse processes are positioned more anteriorly. Laterally, the transverse process is well expanded posteriorly to contact with the associated transverse process of the posterior sacral, enclosing a small fenestra between the two. A similar structure is slightly reduced in the posterior sacral. More laterally, the transverse processes have a poorly ossified articular facet for the ilium. One isolated sacral is preserved near the lower jaws (Fig 1). Its neural arch is exposed posterodorsally. Two transverse processes extend posterolaterally, setting an acute angle of about 45 degrees with the axial column. The prezygapophyses are oriented anterolaterally with nearly vertical articular facets. The neural spine is stout. The neural canal is exposed posteriorly. It is small and enclosed by the neural arch. Postzygapophyses form the posterior margin of the neural arch, just slightly posterior to the oblique transverse processes.

#### Appendicular skeleton

The right scapula and coracoid are preserved in articulation and exposed in lateral view, but they are not fused together as in osteologically mature pterosaurs. The left scapula and coracoid are also preserved but obscured by other elements. The right scapula is 54.5 mm long. Anteriorly, the scapula and coracoid form the glenoid fossa, but the scapula contributes more to the glenoid fossa than the coracoid. Dorsal to the glenoid fossa, the scapula is straight before curving posteroventrally at its proximal third point. The scapular shaft is near constant in width. Posteriorly, the ventral margin of the shaft becomes constricted and convergent with the dorsal margin, forming a rounded and sloped distal end. The distal end of the scapula is incompletely ossified.

The coracoids are partially obscured by overlapping bones. The left coracoid is 47.5 mm long, shorter than the scapula. Below the glenoid fossa, the coracoid bears a fan-like flange that extends posteriorly and continues ventrally. The flange is reduced posteriorly along the coracoid shaft and disappears close to the mid-point of the shaft. Similar flanges are also present in other azhdarchoids from China, and they are comparable with the coracoid flange of azhdarchids, and the coracoid tubercle of tapejarids and thalassodromines from Brazil. Posterior to the flange, the coracoid shaft expands bilaterally. A facet is well developed at the coracoidal terminus for articulating with the sternum.

The sternum is preserved in dorsal view (Fig 5). The cristospine is weakly developed as in tapejarids, such as *Eopteranodon*, *Tapejara*, and *Caiuajara* [41, 45, 46]. The sternal plate is subrectangular and wider than long, different from the square or semicircular shape in tapejarids [41, 45, 47]. The sternal plate is thick anteriorly and thin posteriorly. The posterior margin is well preserved medially, but poorly preserved laterally. Along the lateral margins of the sternal plate, crenulations are developed as possible articular facets for the anterior sternocostae. On the sternal plate, a fossa is present near the cristospine, but it does not perforate the plate to form a pneumatic foramen as in *Tapejara* [41].

The right humerus is preserved separate from the associated ulna and radius. It is exposed in posteroventral view and slightly damaged at the posterior tuberosity and distal end. The left humerus appears to be complete, but it is overlapped partially by the right humerus so that its proximal portion is poorly exposed. The left humerus is 78.6 mm long. The well-developed deltopectoral crest is proximally positioned, extending about 25.4% of the humeral shaft. The crest is elongate, not warped, and slightly curved ventrally with a subrectangular profile. Its straight proximal margin is located at the same proximal level as the humeral head. Anteriorly, the crest is rounded and slightly thickened. It is uncertain whether a pneumatic foramen is present on the ventral surface due to compression. Posterior to the humeral head, however, a possible pneumatic foramen is present on the dorsal surface. The humeral shaft is slightly curved anteriorly and expanded distally. An unfused epiphysis is present and displaced from the left humerus. The main part of the epiphysis is exposed and has a subrectangular shape. It is convex centrally, turning upwards at the proximomedial and distolateral corners. A small element, possibly representing another epiphysis, is situated between the distal end of the right humerus and the ulna.

The ulna and radius are preserved for both sides, but the left side is obscured proximally by the overlapping right side. They are elongate and slender with a length of 112.2 mm for the radius and 113.6 mm for the ulna. The ulna is slightly wider than the radius in diameter. The ulna is expanded proximally and distally, producing a constricted shaft. The proximal end of the ulna is expanded more ventrally, whereas the distal end is expanded more dorsally. The left ulna contacts the radius on its proximal and distal ends. The radius appears to be concave on its proximal end and convex on its distal end.

The carpus is preserved for both sides (Fig 6). The right carpus is visible in dorsal view and articulated with the ulna and radius proximally and metacarpal I and wing metacarpal IV distally. The left carpus is visible in ventral view, but its elements are disarticulated. The carpus is composed of two proximal carpals, two distal carpals, and one preaxial carpal. The two proximal carpals are not fused to form the proximal syncarpal, and the two distal carpals are not fused to form the distal syncarpal of mature pterosaurs. In dorsal view, the radiale is semilunate, and the ulnare is irregular in shape. In ventral view, they are poorly ossified with a rugose surface. In the distal carpal series, the anterior distal carpal is relatively smooth and large. It is distinctly expanded at the anterodistal corner forming a round articular facet. The posterior distal carpal is oval and poorly ossified. Proximally, the preaxial carpal possibly articulates with the anterodistal corner of the anterior distal carpal. A fovea is developed on the preaxial carpal, possibly as the articular facet for sesamoid A in pterosaurs [48]. On the left side, a tiny element is placed against the preaxial carpal, possibly representing the sesamoid A.

The pteroids are disarticulated from their associated carpi. The pteroid is long, slender, and rod-like. It is 70.5 mm in length, about 62% of the ulna length. Proximally, the pteroid is curved and expanded. The shaft of the pteroid is nearly straight and pointed distally.

The four metacarpals vary in length but are distally positioned at almost the same level. Metacarpals I–III are slender, whereas wing metacarpal IV is robust and dominates the metacarpus as in other pterosaurs. The metacarpal I is elongate and contacts the carpus, which is unknown in other chaoyangopterids [5, 9]. By contrast, metacarpals II and III are strongly reduced in length–less than one third of the length of the metacarpal I–and fail to contact the carpus proximally. Distally, metacarpals I–III are expanded more dorsoventrally than anteroposteriorly. The wing metacarpal IV is large with a length of 145.3 mm (left) and 150.1 mm (right), 128–132% of the length of the ulna. The wing metacarpal is broad proximally, constricted along the shaft, and abruptly expanded at the distal end. This distal end is a dorsoventrally oriented trochlea forming a roller joint for articulation with the first wing phalanx.

The manual phalangeal formula is 2-3-4-4-x, typical of pterosaurs. The manual digits I–III are preserved in articulation. The distal portion of the right digit III is broken, but its imprint is still identifiable. On the left side, the ungual of digit II is damaged distally; the phalanx III-3 is completely damaged. The relative length of the digits increase from digit I to digit III. The phalanx III-1 is the longest of the manual phalanges, slightly longer than the phalanx I-1 and much longer than phalanx II-1. This condition is different from *Chaoyangopterus*, in which the phalanx II-1 is subequal with the phalanx III-1 and much longer than the phalanx I-1 [9]. The phalanx II-1 is slightly shorter than the phalanx II-2; in *Chaoyangopterus*, the condition is reversed [9]. The phalanx III-2 is nubbin-like and the shortest of the manual phalanges (Table 1). Except for the phalanx III-2, the non-ungual phalanges are expanded at their proximal end, reduced distally along the shaft, and slightly expanded at the distal condyle. The distal ends are about half as wide as the proximal ends. Ventrally, a distinct concave margin is present and reduced distally along the shaft. The manual unguals are approximately twice as large as the pedal unguals in size. Proximally, the flexor tubercle is well developed at the proximal margin of the ungual. Distally, the ungual is strongly curved and sharply pointed. The curvature is about 70 degrees along the inner margin, which is more curved than the outer margin.

**Table 1:** Measurements of *Jidapterus edentus* (RCPS-030366CY). Length in millimeters.

Left side	Right side
Scapula and Coracoid: 57.5/47.5	Scapula: 54.5
Humerus: 78.6	Metacarpals I-III: 137/39.6/39.6
Ulna and Radius: 113.6/112	Wing metacarpal IV: 150.1
Pteroid: 70	Manual digit I: 18.9/15
Metacarpals I-III: 137/39.6/39.6	Manual digit II: 13.4/14.4/15
Wing metacarpal IV: 145.3	Manual digit III: 21.4/5.2/14/?
Manual digit I: 18/?	Wing digit IV: 171.3/118/72.6/?
Manual digit II: 13.2/13.4/?	Femur: 100.6
Manual digit III: 19.2/?/?/?	Tibia: 145.5
Wing digit IV: 185.4/122.3/74.6/38.3	Pedal digit I: 12/8.2
Femur: 100.6	Pedal digit II: 7.7/10.5/7.3
Tibia: 148.1	Pedal digit III: 9.9/2/10.1/7.7
Metatarsals I-V: 36.5/36.7/34/30.4/10.5	Pedal digit IV: >10/2.2/2.2/7.2/7
Pedal digit I: 12.6/9	Third cervical vertebra: 29.8
Pedal digit II: 7.7/11.4/8.2	Fourth cervical vertebra: 43.2
Pedal digit III: 10.7/2.2/10.3/7	Fifth cervical vertebra: 38.2
Pedal digit IV: 13.4/2/1.7/7.7/7.2	Sixth cervical vertebra: 33.7
Pedal digit V: 2	Seventh cervical vertebra: 32.3

The four wing phalanges of the digit IV sequentially reduce in size. The first wing phalanges are preserved with the right one exposed in dorsal view and the left one in ventral view. The first wing phalanx is the longest element in the wing. Proximally, the first wing phalanx expands anteroposteriorly to nearly twice the diameter of the shaft. The extensor tendon process is positioned anteriorly on the proximal end of the first wing phalanx, and unfused with the latter. Generally, the extensor tendon process fuses with the first wing phalanx in mature stages of pterosaurs [36, 37]. Therefore, the unfused condition implies an immature stage for this specimen. Posterior to the process, the proximal end of the first wing phalanx bears a curved articulation associated with the trochlea of the wing metacarpal. The proximal portion of the first wing phalanx is smooth on the dorsal side, but a large pneumatic foramen is present on the ventral side, which is not reported in other chaoyangopterids [9, 13, 19]. The shaft is smooth, straight, and relatively constant in diameter. The first wing phalanx bears distal end comparable with the shaft in diameter. The distal ends are rounded and articulated with the slightly curved proximal end of the second wing phalanx.

The second wing phalanges are preserved across one another. Proximally, the left phalanx is partially overlapped by the right ulna, and the right one is partially damaged on its shaft. They articulate with the first phalanx proximally, but their distal ends are disarticulated from the succeeding third phalanx. The second wing phalanx is reduced to about 66–69% the length of the first wing phalanx. Proximally, the second wing phalanx expands more posteriorly than anteriorly. The shaft of the wing phalanx is smooth and nearly straight. Ventrally, a round ridge is weakly developed along the middle portion of the shaft, differing from a well-developed ridge creating T-shaped cross-section in azhdarchids [40, 49, 50]. Distally, the phalanx is slightly expanded.

Both third wing phalanges are preserved. The right one is partially overlapped by the mandible. This phalanx is about 40% of the length of the first wing phalanx. In most tapejarids, the third wing phalanx is slightly longer than the half of the first one (Table 2). The shaft of the phalanx is gradually constricted towards the distal end. Its proximal third portion is flat, and the remaining portion is rod-like. The distal end is slightly expanded and more condyle-shaped.

**Table 2:** Measurements and proportions of major limb bones in selected azhdarchoids. Length in millimeters.

**Taxon**	**Hu**	**Ul/Ra**	**Wmc**	**Wp1**	**Wp2**	**Wp3**	**Fe**	**Ti**	**Mt**
***Chaoyangopterus* (IVPP V13397)**	93	133	185	200/199	120	78	133/131	205/208	44/43
***Chaoyangopterus* (PMOL-AR00076)**	78/79	110.5	149.4/151	164/162	112	71/70	107.7/106	158/156	?
***Jidapterus* (RCPS-030366CY)**	78.6/81.3	113.6	145.3/150.1	171.3/185.4	118/122.3	72.6/74.6	100.6	148.1/145.5	34
**Chaoyangopterid (LPM-L112113)**	80	117	155	190	127	?	113	?	?
***Shenzhoupterus* (HGM 41HIII-305A)**	66	105	140	147	100	68	102	139	39
***Zhejiangopterus* (ZMNH M1323)**	137	234	336	322	220	?	222	265	?
***Quetzalcoatlus* sp. (TMM 42422)**	250	358	620	602	305	156	?	604	?
***Tupuxuara leonardii* (IMCF 1052)**	234	291	359	505	301	208	298	398	88.2
***Eopteranodon* (BPV 078)**	63	94	99.5	131	99	?	75	?	?
***Eopteranodon* (D 2526)**	68/69	93/96	105/106	137/134	103/102	75/76	80	117	27
***Eoazhdarcho* (GMN-03-11-002)**	90	122	135	178	139	93	94	160	?
***Tapejara wellnhoferi* (SMNK PAL 1137)**	69.4	101.5	107	144	113.8	?	82.5	114.7	?
***Sinopterus dongi* (IVPP V13363)**	59/58	88/87.5	95	121	91/88	65.5/63	74	104	20.5
***Sinopterus dongi* (D 2525)**	108/109	154	169/170	215	156	106	135/137	185	37
***Huaxiapterus jii* (GMN-03-11-001)**	79	117	132	163/160	127	91/92	100	141	34
***“H*.*” benxiensis* (BXGM V0011)**	62	119	133	176	130	98	112	154	35
***“H*.*” corollatus* (ZMNH M8131)**	79.7	114	152	176	108.5	69.4	92.5	155	31
**Jehol tapejarid (PMOL-AP00007)**	59.5	82	96	120	94	65	63	107	27
**Jehol tapejarid (PMOL-AP00009)**	75	107	108	145	106	?	85	?	?
**Jehol tapejarid (PMOL-AP00011-1)**	106	155	160	?	150	96	?	?	?
**Jehol tapejarid (PMOL-AP00013)**	86	123.5	117	175	127	92	?	?	?
**Jehol tapejarid (PMOL-AP00016)**	58	77	85	104	83.5	59	67.5	96	26
**Jehol tapejarid (PMOL-AP00017)**	62	88	100	125	?	?	76	115	23
**Jehol tapejarid (PMOL-AP00021)**	53	73	?	?	?	?	55	84	22
**Jehol tapejarid (PMOL-AP00022)**	59	82	88	122	82	56	70	99	?
**Jehol tapejarid (PMOL-AP00025)**	51	76	87	114	81	56	60	92	23
**Jehol tapejarid (SDUST-V1016)**	?	?	?	230	184	125	?	?	?
**Jehol tapejarid (SDUST-V1015)**	42	?	69	?	?	?	51	76	19
**Jehol tapejarid (SDUST-V1004)**	73	102	103	134	99.5	?	?	?	?
**Jehol tapejarid (SDUST-V1013)**	54	78	90	107	80	61	61	90	22
**Jehol tapejarid (LPM-L111609)**	70	103	105	137	?	?	83	126	32
**Taxon**	**Hu/Fe**	**Wmc/Hu**	**Wmc/Ul**	**Wmc/Wp1**	**Wp1/Fe**	**Wp1/Wp2**	**Wp1/wp3**	**Ti/Fe**	**Fl/Hl**
***Chaoyangopterus* (IVPP V13397)**	0.7–0.71	1.99	1.39	0.93	1.50–1.52	1.66–1.67	2.55–2.56	1.54–1.59	1.08
***Chaoyangopterus* (PMOL-AR00076)**	0.72–0.75	1.91–1.92	1.35–1.37	0.91–0.93	1.52–1.53	1.45–1.46	2.30–2.31	1.47	?
***Jidapterus* (RCPS-030366CY)**	0.78–0.81	1.85	1.28–1.32	0.81–0.85	1.70–1.84	1.45–1.52	2.36–2.49	1.45–1.47	1.19–1.23
**Chaoyangopterid (LPM-L112113)**	0.71	1.94	1.32	0.85	1.68	1.5	?	?	0.71
***Shenzhoupterus* (HGM 41HIII-305A)**	0.65	2.12	1.33	0.95	1.44	1.47	2.16	1.36	1.11
***Microtuban* (SMNK PAL 6595)**	?	1.81	1.32	0.9	?	1.18	2.12	?	?
***Zhejiangopterus* (ZMNH M1323)**	0.62	2.45	1.44	1.04	1.45	1.46	?	1.19	?
***Quetzalcoatlus* sp. (TMM 42422)**	?	2.48	1.75	1.03	?	1.96	3.78	?	?
***Tupuxuara leonardii* (IMCF 1052)**	0.79	1.53	1.23	0.71	1.69	1.68	2.43	1.34	1.13
***Eopteranodon* (BPV 078)**	0.84	1.57	1.06	0.76	1.75	1.32	?	?	?
***Eopteranodon* (D 2526)**	0.85–0.86	1.54	1.10–1.13	0.77–0.79	1.68–1.71	1.31–1.33	1.76–1.83	1.46	1.19–1.21
***Eoazhdarcho* (GMN-03-11-002)**	0.96	1.5	1.11	0.76	1.89	1.28	1.91	1.7	?
***Tapejara wellnhoferi* (SMNK PAL 1137)**	0.84	1.54	1.05	0.74	1.75	1.27	?	1.39	?
***Sinopterus dongi* (IVPP V13363)**	0.78–0.8	1.61–1.64	1.08–1.09	0.79	1.63	1.33–1.38	1.85–1.92	1.41	1.21–1.22
***Sinopterus dongi* (D 2525)**	0.8	1.56	1.1	0.79	1.57–1.59	1.38	2.03	1.35–1.37	1.21
***Huaxiapterus jii* (GMN-03-11-001)**	0.79	1.67	1.13	0.81–0.83	1.60–1.63	1.26–1.28	1.74–1.79	1.41	1.19
***“H*.*” benxiensis* (BXGM V0011)**	0.55	2.15	1.12	0.76	1.57	1.35	1.8	1.38	1.04
***“H*.*” corollatus* (ZMNH M8131)**	0.86	1.91	1.33	0.86	1.9	1.62	2.54	1.68	1.24
**Jehol tapejarid (PMOL-AP00007)**	0.94	1.61	1.17	0.8	1.9	1.27	1.85	1.7	1.21
**Jehol tapejarid (PMOL-AP00009)**	0.88	1.44	1.01	0.74	1.71	1.37	?	?	?
**Jehol tapejarid (PMOL-AP00011-1)**	?	1.51	1.03	?	?	?	?	?	?
**Jehol tapejarid (PMOL-AP00013)**	?	1.36	0.95	0.81	?	1.38	1.9	?	?
**Jehol tapejarid (PMOL-AP00016)**	0.86	1.47	1.1	0.82	1.54	1.25	1.76	1.42	1.16
**Jehol tapejarid (PMOL-AP00017)**	0.82	1.53	1.14	0.8	1.64	?	?	1.51	1.16
**Jehol tapejarid (PMOL-AP00021)**	0.96	?	?	?	?	?	?	1.53	?
**Jehol tapejarid (PMOL-AP00022)**	0.84	1.49	1.07	0.72	1.74	1.48	2.17	1.41	?
**Jehol tapejarid (PMOL-AP00025)**	0.85	1.71	1.14	0.76	1.9	1.41	2.04	1.53	1.22
**Jehol tapejarid (SDUST-V1016)**	?	?	?	?	?	1.25	1.84	?	?
**Jehol tapejarid (SDUST-V1015)**	0.82	1.64	?	?	?	?	?	1.49	?
**Jehol tapejarid (SDUST-V1004)**	?	1.41	1.01	0.77	?	1.35	?	?	?
**Jehol tapejarid (SDUST-V1013)**	0.89	1.67	1.15	0.84	1.75	1.34	1.75	1.48	1.28
**Jehol tapejarid (LPM-L111609)**	0.84	1.5	1.02	0.77	1.65	?	?	?	1.15

Abbreviations: Fe, femur; Fl, forelimb (humerus+ulna+wing metacarpal); Hl, hindlimb (femur+tiba+metatarsal III); Hu, humerus; Mt, metatarsal III; Ra, radius; Ti, tibia or tibiotarsus; Ul, ulna; Wmc, wing metacarpal; Wp1, first wing phalanx; Wp2, second wing phalanx; Wp3, third wing phalanx. Measurements from the literature (Cai & Wei [38]; Eck, Elgin & Frey [40]; Elgin & Frey [51]; Lü & Ji [11]; Lü& Yuan [46]; Lü & Zhang [10]; Lü et al. [4]; Lü et al. [41]; Lü et al. [44]; Lü et al. [52]; Lü et al. [53]; Martill et al. [54]; Unwin, Lü & Bakhurina [55]; Wang & Zhou [8]; Wang & Zhou [56]; Zhou [18]).

The fourth wing phalanx is preserved on both sides. However, the right fourth phalanx is broken and rotated at its midpoint, and its proximal end is hidden by the overlapping tibia. On the left side, the terminal end is broken and slightly displaced from its original position. The fourth wing phalanx is reduced in size and much smaller than other wing phalanges. The phalanx is slender, rod-like, and distinctly expanded at the proximal end with a shallow cotyle-shaped articular facet. Distally, the phalanx is constricted along shaft. The terminal ends are poorly ossified.

The probable left pelvic girdle is preserved in association in medial view (Fig 5). The acetabulum is invisible in medial view and appears to be imperforate, as in other pterosaurs. The ilium is obscured posteriorly by overlapping of the right femur, and the preacetabular process is missing. In contrast, the main body of the ilium is exposed. It starts narrow anteriorly and gradually deepens posteriorly along the iliopubic suture, reaching the maximum depth of about 10.4 mm at the conjoined point of the ilium, pubis, and ischium. Posterior to this conjoined point, the ilium is reduced along the ilioischial suture. The pubis is slightly displaced from its original position. It is a laminar plate with a thick anterodorsal edge bordering the acetabulum. Along the acetabulum, the pubis is thickened and extended anterodorsally, well beyond the anterior margin of the pubic plate, forming a distinct concave anterior margin of the pubis with the latter. This condition is different from a straight or slightly concave anterior margin of the pubis in other azhdarchoids [41, 57, 58]. Ventrally, the pubic plate seems to extend anteroventrally and leaves a distinct space with the ischium ventrally. The pubic plate is subrectangular with parallel anterior and posterior sides and a round ventral margin. The pubic plate is much anteroposteriorly shorter than it is dorsoventrally deep (Fig 5). In contrast, the pubic plate of *Tapejara wellnhoferi* Kellner 1989 [59] is elongated anteroposteriorly with ventrally divergent anterior and posterior margins. A round obturator foramen is present on the posterior edge of the pubic plate. The foramen is open posteriorly, implying that the ischium contributes to enclose the foramen with the pubis. The left ischium is partially exposed. It articulates with the ilium dorsally and the pubis anterodorsally. Ventrally, the pubis and ischium seem to be separated. A possible separation between the pubis and ischium is also known in *Vectidraco daisymorrisae* Naish et al. 2013 [57] but absent in other known azhdarchoids [41, 58]. However, this ventral separation is also affected by ontogenetic changes of the pubis and ischium [58, 60]. Another large and lamina-shaped element possibly represents the right ischium, but it is partially obscured by the left ischium.

In contrast, the right pelvic girdle is poorly identified and scattered. The postacetabular process of the right ilium is isolated and located next to the atlas-axis complex, while the right pubis is identified near the left coracoid. The postacetabular process is exposed in lateral view with a smooth surface. It is robust and hatchet-like (Fig 3). Anteriorly, its neck portion is narrow and has parallel margins with a depth of 5.4 mm. This structure is comparable with that of *Vectidraco* [57], but different from a constricted condition in other azhdarchoids [58]. Posteriorly, as in other azhdarchoids, it is hatchet-like with a pointed dorsal process and a more robust ventral process that measures 17 mm along the lateral margin. The pointed dorsal process is distinctly less than that of other azhdarchoids [41, 58]. The right pubis is perforated by an obturator foramen as the left one (Fig 1).

Both hind limbs are nearly complete with the femora, tibiae and fibulae, and pedes preserved. The femora are preserved side by side (Figs 1 and 5), partially overlapped by the sternum and the skull. The right femur is exposed in posterior view, whereas the left one is exposed in medial view. Proximally, the femoral head is condyle-shaped and separated from the shaft by a distinct neck. As in other pterodactyloids, the femoral head extends dorsomedially and is located on the dorsomedial side, rather than the medial side of the shaft. In posterior view, the greater trochanter forms a round corner of the proximal shaft. In addition, the shaft has a pneumatic foramen near its proximal margin. The femoral shaft is slightly curved. The distal articulation is formed by two condyles (Fig 4D). Both condyles are comparable in size, but the medial condyle extends more caudally than the lateral one. They are widely separate with a distinct intercondylar sulcus.

The tibia is 148.1 mm long, the longest element in the hindlimb, but distinctly shorter than the first wing phalanx (Table 1). The similar condition is known in *Chaoyangopterus* (PMOL-AP00076; [19]); whereas, in the relatively larger holotype of *Chaoyangopterus* (IVPP V13397), the tibia is the longest of the long bones [9]. This difference in *Chaoyangopterus* is possibly due to the ontogenetic variance. Proximally, the tibia is broad and gradually constricts along the shaft towards the distal end. The distal end of the tibia is not fused with proximal tarsals as in osteologically mature pterosaurs [36, 37].

The fibula is reduced to a slender and rod-like element. The left fibula is obscured by the overlapping right femur and tibia. The right fibula is well exposed with a length of 61.2 mm. Its proximal end is not positioned at the level of the tibia proximal end, possibly due to preservation. However, the similar condition seems to be present on the left side.

The tarsus is preserved on the left side. Its elements are slightly displaced from their original position, although they are still preserved between the tibia and pes. The tarsus is composed of two proximal tarsals and two distal tarsals. The proximal tarsals are not fused with the tibia. They are large and semilunate, which functions as a roller joint to articulate with the distal tarsals. Lateral distal tarsal is subrectangular and located distally against metatarsals III–V. The medial distal tarsal is irregular in shape with a tubercle-like process on its proximomedial corner. It is preserved against metatarsals I–II distally, the lateral distal tarsal laterally, and the proximal tarsals proximally.

The pes is preserved in articulation on both sides (Fig 7). The metatarsus is tightly bound. Metatarsals I–IV are all long and slender. Metatarsals I and II are subequal in length, whereas metatarsals III and IV are successively shorter. In *Chaoyangopterus*, however, metatarsal I is shorter than metatarsal II [8]. Metatarsals I and IV appear to be wider than metatarsals II and III in diameter. Metatarsal V is strongly reduced in size, less than one third the length of the other metatarsals (Table 1). Proximally, metatarsal V is expanded with a broad articular facet for the lateral distal tarsal. Distally, the metatarsal V is strongly reduced with pointed but rounded distal end.

The pedal formula is 2-3-4-5-1. The relative length of the digits increases sequentially from digit I to IV. Most of the non-ungual phalanges are elongate, slender, and nearly straight, except for the phalanges III-2, IV-2, and IV-3, which are well reduced and nubbin-like as in other monofenestratan pterosaurs [61]. Proximal phalanges are relatively longer than other pedal phalanges, except for phalanx II-1 that is shorter than II-2. The longest pedal phalanx is phalanx IV-1, which is only slightly longer than the phalanx I-1. Digit V has a single nubbin-like phalanx, typical of pterodactyloids. The pedal unguals are exposed in lateral view and are much smaller than those of the manus. The unguals are laterally compressed, slightly curved, and sharply pointed. Proximally, the flexor tubercle is weakly developed at the base, different from the well-developed tubercles in “*Nemicolopterus*”, *Sinopterus*, and *Tapejara* [52, 62, 63]. Distally, the ungual curves slightly (inner and outer curvatures are 64 and 57.6 degrees, respectively) and points sharply, differing from tightly curved unguals in tapejarids and possibly in thalassodromines (e.g. *Sinopterus*,”*Nemicolopterus*”, and *Tapejara*) [3, 52, 62–65]. The ungual of pedal digit I is slightly larger relative to those of digits II–IV.

### Phylogenetic results

**Fig 8:**
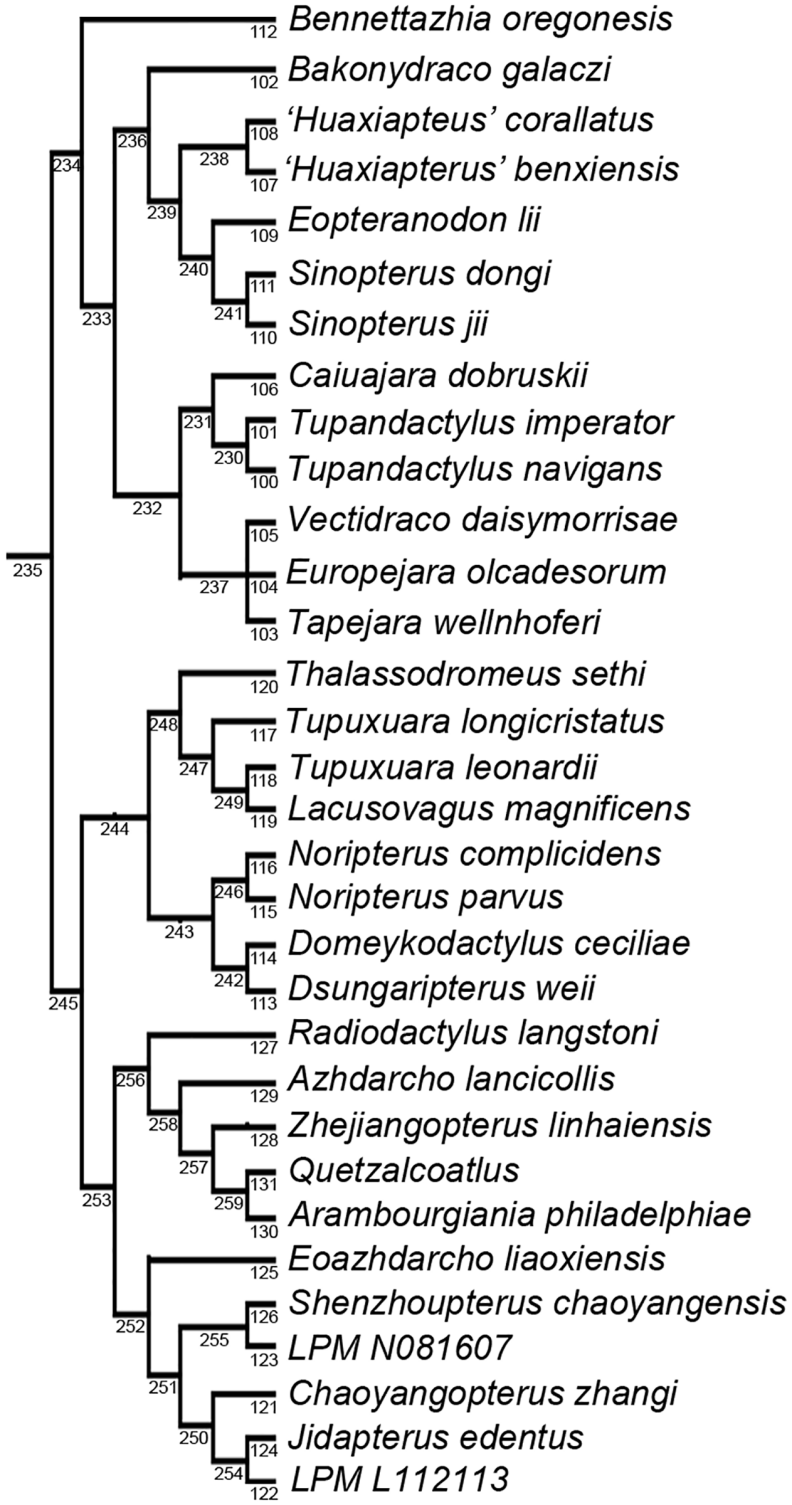
Cladogram depicting the evolutionary relationships of the Azhdarchoidea from the phylogenetic analysis of *Jidapterus edentus* and the Pterosauria. TNT node numbers are depicted below the branches that subtend the nodes. Branch lengths and support measures are listed in S1 Table.

The phylogenetic analysis of *Jidapterus edentus* and the relationships of the Pterosauria resulted in a single most parsimonious tree, depicted in Fig 8 and S3 Fig. These results closely match Zhou et al. [22] and previous versions with the main exception of the new species added. Other changes include rearrangements of anurognathid and tapejarid species, *Normannognathus wellnhoferi* Buffetaut et al. 1998 [66] recovered with *Cycnorhamphus suevicus* Quenstedt 1855 [67], *Ardeadactylus longicollum* von Meyer 1854 [68] placed as sister group to the Gnathosaurinae, pteranodontids recovered with nyctosaurids in a basal pteranodontoid clade, the Lonchodectidae and Boreopteridae are sister taxa, *Guidraco venator* Wang et al. 2012 [69] as sister group to *Ludodactylus sibbicki* Frey et al. 2003 [70], and a *Cearadactylus atrox* Leonardi and Borgomanero 1985 [71]—*Brasileodactylus araripensis* Kellner 1984 [72] sister group is supported (S3 Fig).

*Jidapterus* was recovered as the sister group to the specimen LPM L112113, supporting the hypothesis that *Jidapterus edentus* and *Chaoyangopterus zhangi* are distinct species. It is a possibility that all three taxa could belong to one large and variable species, but *Jidapterus* and LPM L112113 have a closer relationship to the exclusion *Chaoyangopterus*, which would satisfy many of the phylogenetic definitions of a species [73]. In addition, LPM N081607 was recovered as the sister group to *Shenzhoupterus*. The taxonomy of the two Liaoning Paleontological Museum of Chaoyang National Geopark specimens (S1 and S2 Figs) should be the subject of future inquiry, but they can be confirmed as chaoyangopterids. The other relationships of the Chaoyangopteridae corroborate previous results. *Jidapterus* and *Chaoyangopterus* are more closely related to each other than *Shenzhoupterus*, and all three are more closely related to one another than *Eoazhdarcho* in the Chaoyangopteridae. *Eopteranodon* is again recovered as a tapejarid. *Lacusovagus* was recovered in the Chaoyangopteridae by an unpublished analysis mentioned in Witton [20], but here it is placed within *Tupuxuara*. *Tupuxuara* and *Lacusovagus* are known exclusively from the Santana Group of Brazil and chaoyangopterids are known exclusively from the Jiufotang Formation of China, and so this does correspond with the geographic and temporal distributions of these groups.

## Discussion

### Taxonomy

In the Jehol Biota, the five taxa were previously assigned to Chaoyangopteridae, *Chaoyangopterus*, *Jidapterus*, *Shenzhoupterus*, *Eopteranodon*, and *Eoazhdarcho*. Of these, *Chaoyangopterus*, *Jidapterus*, and *Shenzhoupterus* are similar in having a long and low rostrum with a high RI (3.7–5.1), a slightly curved dorsal margin with a straight ventral margin, a long dentary symphysis over half of the mandible length, absence of dentary crest, moderately elongate mid-cervical vertebrae (ratio of the length versus the width is 3–4), and several similar limb proportions (Table 2; see Discussion below), which are characteristic for the Chaoyangopteridae. In contrast, *Eopteranodon* is probably a representative of the Tapejaridae (sensu Kellner and Campos [74]) with which it shares a dentary crest and similar limb proportions (e.g. Wmc/Hu, Wmc/Ul, Wmc/Wp1, Wp1/Wp2, Wp1/Wp3; Table 2), which is consistent with previous phylogenetic analyses [5, 7, 8, 15, 16] and the analysis in this study. *Eoazhdarcho* is currently recovered as a chaoyangopterid [8] and also by the phylogenetic analysis in this study, but has a shorter dentary symphysis (less than half of the mandible length) and similar limb proportions (e.g. Wmc/Hu, Wmc/Ul, Wp1/Wp3) with tapejarids (Table 2).

In the Chaoyangopteridae, *Jidapterus* was argued to be a junior synonym of *Chaoyangopterus* by Wang and Zhou [14], but without providing an explicit justification. *Jidapterus* is more similar to *Chaoyangopterus* than to *Shenzhoupterus* in sharing a general cranial profile and postcranial dimensions, but due to lack of published information about of their holotypes, the differences between *Jidapterus* and *Chaoyangopterus* have been obscured. For example, the manual phalanx II-1 is much shorter than the other proximal phalanges in *Jidapterus*, much different from the original description of the holotype of *Chaoyangopterus* in which the manual phalanx II-1 is the longest [9]. We compared *Jidapterus* with both juvenile and larger specimens of *Chaoyangopterus*. Their features more confidently to exclude *Jidapterus* from *Chaoyangopterus*, such as a shallow longitudinal trough along the labial margin of the rostrum (see Discussion below), a larger angle between the dorsal and postorbital processes of the jugal, the fourth–seventh cervical vertebrae are gradually decreased in length.

Furthermore, *Jidapterus* also differs from *Shenzhoupterus* (RI: 3.7) in having a more elongate rostrum (RI: 5.1), straight occlusal margin of the rostrum, shallow longitudinal trough along the labial margin of the rostrum, length of the fourth to seventh cervical vertebra sequentially decreased, and different limb bone proportions (e.g. Wmc/Hu, Wp1/Fe, Wp1/Wp3; Table 2). In light of these differences between *Jidapterus* and other chaoyangopterids and the results of the phylogenetic analysis, our study supports the validity of *Jidapterus edentus* as a distinct species [8, 13, 15].

Moreover, our study reveals more complete information of *Jidapterus*, such as a sternal plate wider than long, pneumatic foramen on first wing phalanx, hatchet-like postacetabular process with unconstructed neck and small dorsal process, distinctly concave anterior margin of pubis, pubic plate much deeper than long, and the morphology of the pedal phalanges that are unknown in *Chaoyangopterus* and *Shenzhoupterus*. These features have potential to further differentiate *Jidapterus* from the other two, considering that they are somewhat varied in other azhdarchoids [41, 45, 46, 57, 58].

Therefore, the validity of *Jidapterus* is supported by its anatomy and the phylogenetic relationships, although it could be challenged by more complete materials of chaoyangopterids in the future study.

### Osteological comparisons

Redescription of *Jidapterus* reveals more information about the morphology of the chaoyangopterids, such as a concave dorsal margin of the rostrum, a wide sternum plate, an elongate metacarpal I, pelvic girdle and pedal digits. Moreover, the Chaoyangopteridae share with the Azhdarchidae a high RI value and reduced, mildly curved pedal unguals. The long and low rostrum of chaoyangopterids differs from the shorter rostrum found in tapejarids but exhibits similar morphology to the rostra of azhdarchids. The RI values in *Jidapterus* (5.1), *Chaoyangopterus* (4.8–5.3), and *Shenzhoupterus* (3.7) are much higher than in the tapejarids (0.51–0.65) and thalassodromines (1.65–2.45). RI values of chaoyangopterids fall well into the RI range of azhdarchids (4.36 in *Zhejiangopterus* and 7.33 in *Quetzalcoatlus*) [38].

The rostrum is dorsally concave in *Jidapterus*, which is slightly more pronounced than in the rostra of *Chaoyangopterus* and *Shenzhoupterus* [5, 9, 19]. In contrast, the rostrum is nearly straight along the dorsal profile in thalassodromines and azhdarchids, except for *Azhdarcho* in which a dorsally concave rostrum is possibly present [50]. In tapejarids, the dorsal profile of the rostrum is deepened by the well-developed anterior crest of the premaxilla. Among azhdarchoids, the curved dorsal profile of the rostrum is probably diagnostic for the Chaoyangopteridae, although a dorsally concave rostrum is present in other pterosaur groups, including rhamphorhynchids, ctenochasmatoids, and pteranodontians.

Moreover, along the labial margin of the rostrum, there is a shallow longitudinal trough that is dorsally bordered by a low ridge, possibly diagnostic for *Jidapterus*. This labial margin of the rostrum was interpreted by Lü et al. [13] as a ‘midline ridge along the ventral surface of the premaxilla’, and comparable with the ‘median palatal ridge’ of the thalassodromine *Tupuxuara* [38, 74]. In *Tupuxuara leonardii*, the ‘median palatal ridge’ is distinctly ventral to the labial margin of the rostrum, visible in lateral view, and positioned more posteriorly close to the anterior margin of the nasoantorbital fenestra [38, 74, 75]. In contrast, the structure of *Jidapterus* is very elongate, nearly occupies the whole length of the rostrum, and also forms the labial margin of the rostrum. A low condition of the ‘median palatal ridge’ is also known in *T*. *longicristatus*, but is invisible in lateral view. Moreover, the ‘median palatal ridge’ of *Tupuxuara* is flatten laterally; whereas, the labial margin is raised laterally by a low ridge in *Jidapterus*. Therefore, this structure is not the ‘median palatal ridge’ of the thalassodromine *Tupuxuara*. In fact, the ‘median palatal ridge’ is widely absent in tapejarids, chaoyangopterids, and azhdarchids [3, 18, 20, 38, 74]. Although the rostrum is also well known in *Chaoyangopterus* and *Shenzhoupterus*, the shallow trough is not reported, possibly representing a feature characteristic of *Jidapterus*.

Except for a fragment in *Chaoyangopterus* [9], the sternum is only found in *Jidapterus* of all chaoyangopterids. As in other azhdarchoids, the cristospine is short. However, the sternum plate appears to be much wider than long, differing from a square-like or semicircular profile in tapejarids [41, 45, 46]. A square-like sternum is reported in *Eopteranodon* [45], which was first assigned to the Chaoyangopteridae [5] but later referred to the Tapejaridae [7, 8, 15, 16]. Therefore, it remains uncertain whether morphology of the sternum plate in *Jidapterus* is diagnostic in the Chaoyangopteridae.

The metacarpal I of *Jidapterus* is elongate and contacts the distal syncarpal. This feature is unknown in other chaoyangopterids, possibly due to poor preservation. However, *Shenzhoupterus* has a reduced metacarpal I [5]. This variation is difficult to identify as a taxonomic feature, because a slender metacarpal I would be damaged more frequently as a taphonomic artifact. A similar ambiguous condition is also known in some Chinese tapejarids; for example, an elongate metacarpal I is present in *Huaxiapterus jii* [47] but absent in *Sinopterus dongi* and “*Huaxiapterus*” *benxiensis* [42, 53, 56].

The pelvic girdle documented in *Jidapterus*, is the first for the Chaoyangopteridae. As in other azhdarchoids, the pelvic girdle is notable in having a hatchet-like postacetabular process and a broad pubis anterior to the acetabulum [41, 57, 58]. Apart from these, the pelvic girdle shows several distinct features in *Jidapterus*, such as a postacetabular process bearing an unconstricted neck and small dorsal process, concave anterior margin of the pubis, and subrectangular pubic plate with nearly parallel anterior and posterior sides [41, 57, 58]. Among the azhdarchoids, the postacetabular process of *Jidapterus* is comparable with *Tapejara* and *Vectidraco* in having a relatively elongated and unconstricted neck, but it differs from the latter two in having a small dorsal process. In contrast, the neck of the postacetabular process is short and distinctly constricted in other azhdarchoids [58]. Furthermore, the dorsal process of the postacetabular process is smaller than those of other azhdarchoids, possibly typical for *Jidapterus*. The pubis of *Jidapterus* has a concave anterior margin, as in *Vectidraco*, but different from the straight anterior margin in other azhdarchoids [41, 58]. The pubic plate is subrectangular with parallel anterior and posterior sides in *Jidapterus*, while a ventrally widen condition is present in *Tapejara* [41], but it is uncertain in *Vectidraco* and other azhdarchoids [57, 58]. Therefore, the pelvic girdle appears to be more varied in azhdarchoids than the previously realized, although it is still poorly documented.

Among azhdarchoids, the pedal morphology is well known in tapejarids (SMNK PAL 3830, D2525)[3, 52]. In SMNK PAL 3830, the pedal digit III is about 93.8% of the metatarsal III. In contrast, the pedal digit III of *Jidapterus* is relatively shorter, about 85.9% of the metatarsal III. When the ungual is excluded, the ratio of the pedal digit III to the metatarsal III is about 61.5% in SMNK PAL 3830 and 68.2% in *Jidapterus*. This variance is due to a larger ungual in the tapejarids. Moreover, in tapejarids (SMNK PAL 3830, D2525), the penultimate phalanges are much longer than the proximal phalanges in pedal digits II and III; even in the pedal digit IV, the pedal digit IV-1 and IV-4 are subequal in length. In *Jidapterus*, however, a longer penultimate phalanx is only present in pedal digit II, a slightly shorter condition is present in pedal digit III, and a much shorter condition is present in pedal digit IV. In addition, the pedal digit I-1 is the longest in tapejarids, whereas it is slightly shorter than pedal digit IV-1 in *Jidapterus*. The relatively larger unguals and longer penultimate phalanges in tapejarids imply a stronger grasping ability than that in *Jidapterus* or other chaoyangopterids.

**Fig 9:**
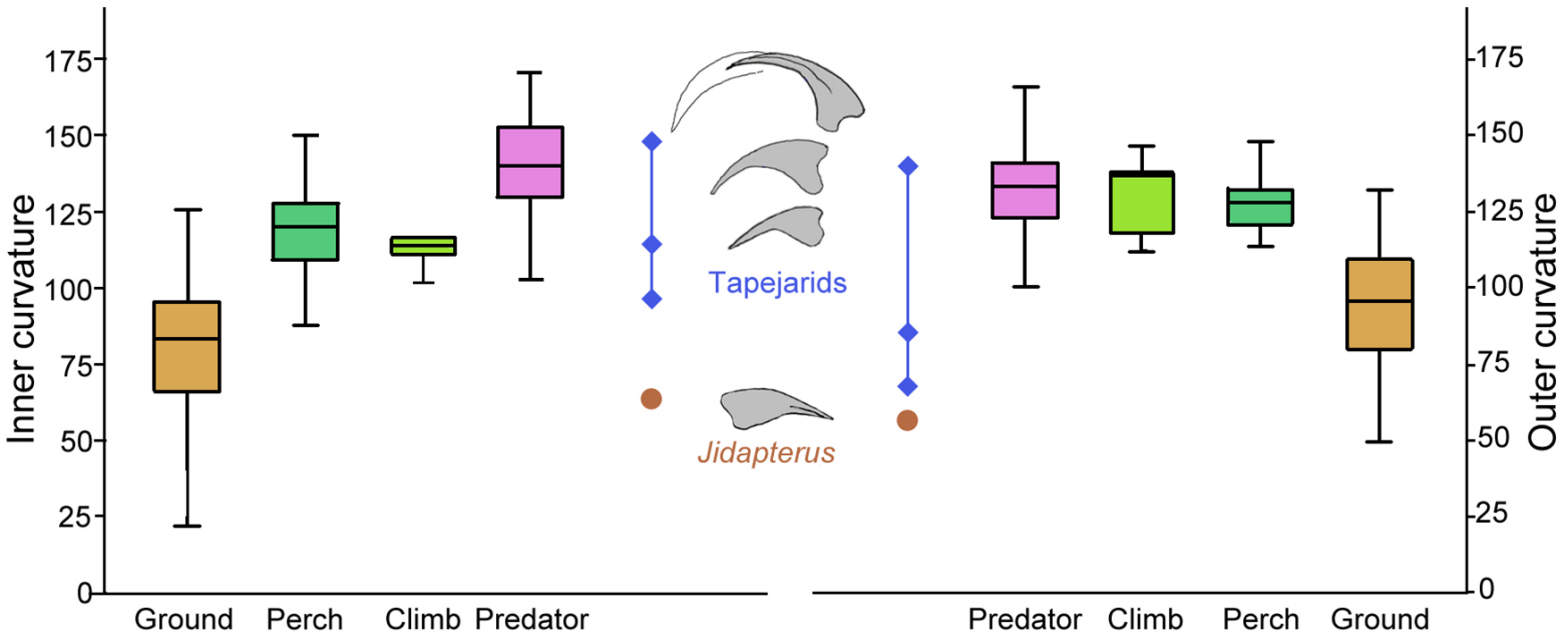
Box plots for the ungual curvature of pedal digit III of azhdarchoids and extant tetrapods. The box plots performed by PAST 2.12 [77], and the extant data from Birn-Jeffery et al. [78].

As in most other pterosaurs, the manual and pedal unguals of *Jidapterus* are disparate in size (the former is roughly twice as large as the latter) and shape. The manual unguals are large and strongly recurved, possibly suitable for the grasping, but the pedal unguals are reduced in size and curvature, possibly implying terrestrial adaptation. The pedal unguals also vary in curvature in other azhdarchoids [39, 52, 62– 64]. The unguals of azhdarchids (e.g. *Zhejiangopterus*) are similar to that of *Jidapterus* with a slight curvature and small size [39, 76]. The curvature value of *Jidapterus* (64 and 57.6 degrees in the inner and outer curvature respectively) is comparable to that of the extant ground tetrapods (Fig 9). However, a tight curvature is present in the pedal unguals of tapejarids (e.g., *Tapejara*, *Sinopterus*, and “*Nemicolopterus*”[3, 41, 62–65]) that possibly have a strong grasping ability (Fig 9) [62, 65]. In *Sinopterus* (D 2525), the ungual is 95.5 and 66.7 degrees in the inner and outer curvature. In the Brazil tapejarid (SMNK PAL 3830), both the ungual and keratinous sheath are well preserved, with larger inner and outer curvature (114 and 84.5 degrees, and 147.5 and 140 degrees). It should be noted that keratinous sheaths are not preserved in *Jidapterus* and there is possible variation between unguals and sheaths. The value of the tapejarids range from 95.5 degrees to 147.5 degrees in the inner curvature, and 66.7–140 degrees in the outer curvature. This range is beyond that of the extant ground tetrapods, and comparable with those of other extant tetrapods in arboreal, climbing, and predatory behavior (Fig 9).

**Fig 10:**
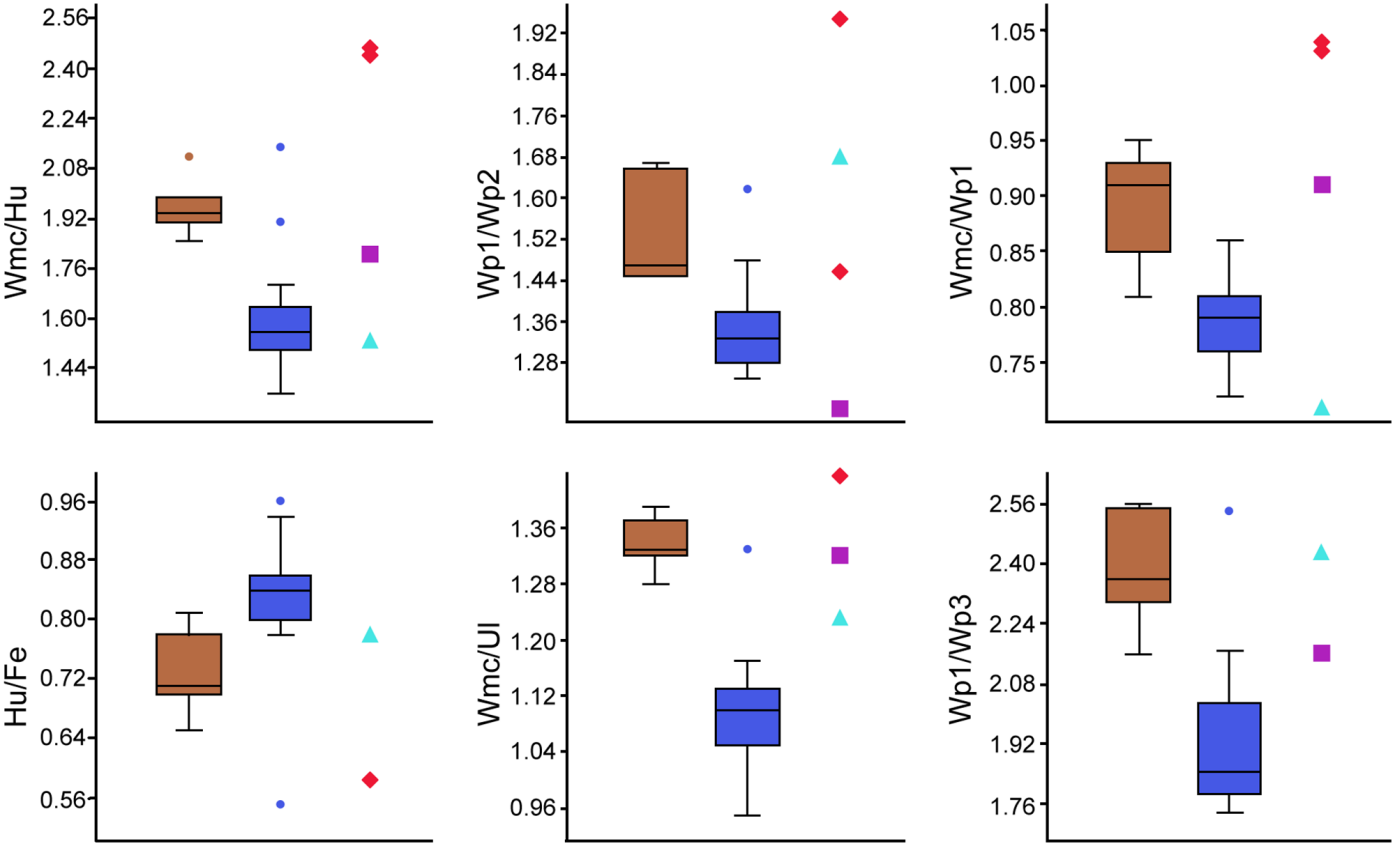
Box plots for the limb proportions of azhdarchoids. The box plots performed by PAST 2.12 [77], and data from Table 2. Chaoyangopterids (brown), tapejarids (dark blue), *Microtuban* (purple square), azhdarchids (red diamond), and *Tupuxuara* (light blue triangle).

In limb proportions, chaoyangopterids are more comparable with azhdarchids than tapejarids (Fig 10, Table 2). For example, the wing metacarpal in chaoyangopterids is elongate and about twice long as the humerus (1.85–2.12). This proportion is slightly higher in azhdarchids (2.45–2.48) [51] but lower in tapejarids and thalassodromines (1.39–1.67) (except for “*Huaxiapterus*” *corollatus* (2.15) and “*H*.” *benxiensis* (1.91)). The ratio of wing metacarpal to ulna is 1.28–1.39 in chaoyangopterids, 1.44–1.75 in the azhdarchids, and 1.05–1.13 in tapejarids (except for “*H*.” *corollatus* with 1.33). The ratio of wing metacarpal to the first wing phalanx is 0.91–0.95 in most chaoyangopterids (except for *Jidapterus* with 0.81–0.85), 1.03–1.04 in the azhdarchids, and 0.74–0.86 in tapejarids and 0.71 in thalassodromines. The ratio of humerus to femur is 0.62–0.75 in chaoyangopterids (except *Jidapterus*: 0.78–0.81), 0.62 in *Zhejiangopterus*, and 0.78–0.96 in tapejarids (except “*Huaxiapterus*” *benxiensis*: 0.55). The first wing phalanx versus the femur in length is about 1.44–1.53 in chaoyangopterids (except *Jidapterus*: 1.70–1.84) and *Zhejiangopterus* but larger in tapejarids (1.57–1.9). The ratio of the first to second wing phalanges varies in the range of 1.45–1.67 in chaoyangopterids, 1.67 in thalassodromines, and 1.46–1.96 in azhdarchids, but smaller in tapejarids (1.26–1.38) (except “*H*.” *corollatus*: 1.62). The ratio between the first and third wing phalanges is 2.16–2.56 in chaoyangopterids, 3.78 in *Quetzalcoatlus*, and 1.74–2.03 in tapejarids (except “*H*.” *corollatus*: 2.54).

Based on the above limb proportions, *Eopteranodon* falls well into the Tapejaridae, which is consistent with their cranial morphology and previous phylogenetic analyses [7, 8, 15, 16]. These possible differences between chaoyangopterids, tapejarids, and thalassodromines are potentially useful for identifying their skeletons when lacking cranial morphology. For instance, two disarticulated individuals (A and B) can be identified in *Tapejara wellnhoferi* specimen SMNK PAL 1137 based on repeated and different-sized limb bones, whereas the remaining elements of the skeleton could not be referred to individual A or B [41]. The unreferred limb bones and the bones of individual B bear comparable proportions to other tapejarids (Table 2), implying that they may belong to the same specimen. Moreover, these differences imply a possible affinity of the non-azhdarchid *Microtuban* with the chaoyangopterids or thalassodromines [51]. As shown in Table 2, the limb proportions of *Microtuban* are more comparable to that of the chaoyangopterids than the thalassodromines.

Both the juvenile specimens of *Jidapterus* (RCPS-030366CY) and *Chaoyangopterus* (PMOL-AR00076) are comparable in length on nearly all their forelimb bones (except the first wing phalanx, Table 2). However, the major ratios between the various limb bones (such as Hu/Fe, Wmc/Hu, Wmc/Ul, Wmc/Wpc, Wp1/Fe, Table 2) of the two specimens are distinctly different. Meanwhile, these ratios are quite similar in both smaller (PMOL-AR00076) and larger (IVPP V13397) specimens of *Chaoyangopterus*, which indicate that the limb bone proportions are relatively stable ontogenetically, and also support that *Jidapterus* is not a juvenile form of *Chaoyangopterus*. In addition, proportions of wing phalanges (Wp1/Wp2 and Wp1/Wp3) are comparable in *Jidapterus* (RCPS-030366CY), *Chaoyangopterus* (PMOL-AR00076) and *Shenzhoupterus* (HGM 41HIII-305A), indicating that these chaoyangopterids may have the same pattern of ontogenetic feature of wing phalanges.

### Paleoecological implications of chaoyangopterids

The azhdarchoid pterosaurs, especially the huge azhdarchids, are predicted to have an effective terrestrial capability from their long limbs and compact feet [3, 65, 76]. Additional evidence of terrestrial adaptations in azhdarchids includes small manual digits, pes, unguals, and possible footprints [3, 76]. In contrast, the pedal unguals are poorly known in other medium-sized azhdarchoids, except for the tapejarids and thalassodromines, in which the sharp and well curved unguals are possibly associated with grasping trees [62, 65], as plotted in Fig 9. Among chaoyangopterids, *Jidapterus* possibly exhibits terrestrial adaptations as in azhdarchids, because the pedal unguals are greatly reduced and gently curved, and comparable with that of the extant ground tetrapods in curvature (Fig 9). In the Jehol Biota, the putative arboreal tapejarids are dominant in the Jiufotang Formation, co-existing with arboreal avians and feathered dinosaurs [79, 80]. In contrast, the rarity of the chaoyangopterids suggests that a terrestrial lifestyle may have limited their diversity in the forest-dominated ecosystem of the Jehol Biota.

Chaoyangopterids were originally thought to be primarily fish-eaters with long jaws [14]. However, recent studies of Witton and Naish [76] and Witton [3] have argued that chaoyangopterids had a low-profile skull with a long beak similar to putatively carnivorous azhdarchids, and are possibly more or less omnivorous with a varied diet including small prey or carrion. This feeding style of the chaoyangopterids would be consistent with their relatively terrestrial adaptations [3, 76]. Compared with chaoyangopterids, tapejarids have a parrot-like beak–suitable for feeding on fruits or seeds [2, 3, 14, 79–82]–that may be associated with their tree-grasping abilities [62, 65]. However, other diets are possibly involved as in extant seed-eating birds [3]. Lifestyle differences of chaoyangopterids and tapejarids may reveal ecomorphic disparities in the azhdarchoids of the Jehol Biota.

## Supporting information

S1 Appendix A TNT executable file. (TNT)

S1 Fig A undescribed chaoyangopterid (LPM-L112113). An incomplete skeleton includes skull, lower jaws, forelimbs and partial hindlimbs, collected from the Jiufotang Formation of Shangheshou, Chaoyang, western Liaoning. Scale bar = 10 mm.
(TIF)

S2 Fig Rostrum of the undescribed chaoyangopterid (LPM-N081607) from the Jiufotang Formation of Shangheshou, Chaoyang, western Liaoning. (TIF)

S3 Fig Single most parsimonious cladogram from the phylogenetic analysis of *Jidapterus edentus* and the relationships of Pterosauria. TNT node numbers are depicted below the branches that subtend the nodes. Branch lengths and support measures are listed in S1 Table.
(TIF)

S1 Table Branch lengths as well as Bremer, Bootstrap, and Jacknife scores for the node numbers from the phylogenetic analysis in TNT shown in S3 Fig. (XLSX)
