# Supplementary figures and images for: The toothless pterosaur Jidapterus edentus (Pterodactyloidea: Azhdarchoidea) from the Early Cretaceous Jehol Biota and its paleoecological implications
Source: PLoS One. 2017 Sep 26;12(9):e0185486. doi: 10.1371/journal.pone.0185486 (PMC5614613; doi:10.1371/journal.pone.0185486)

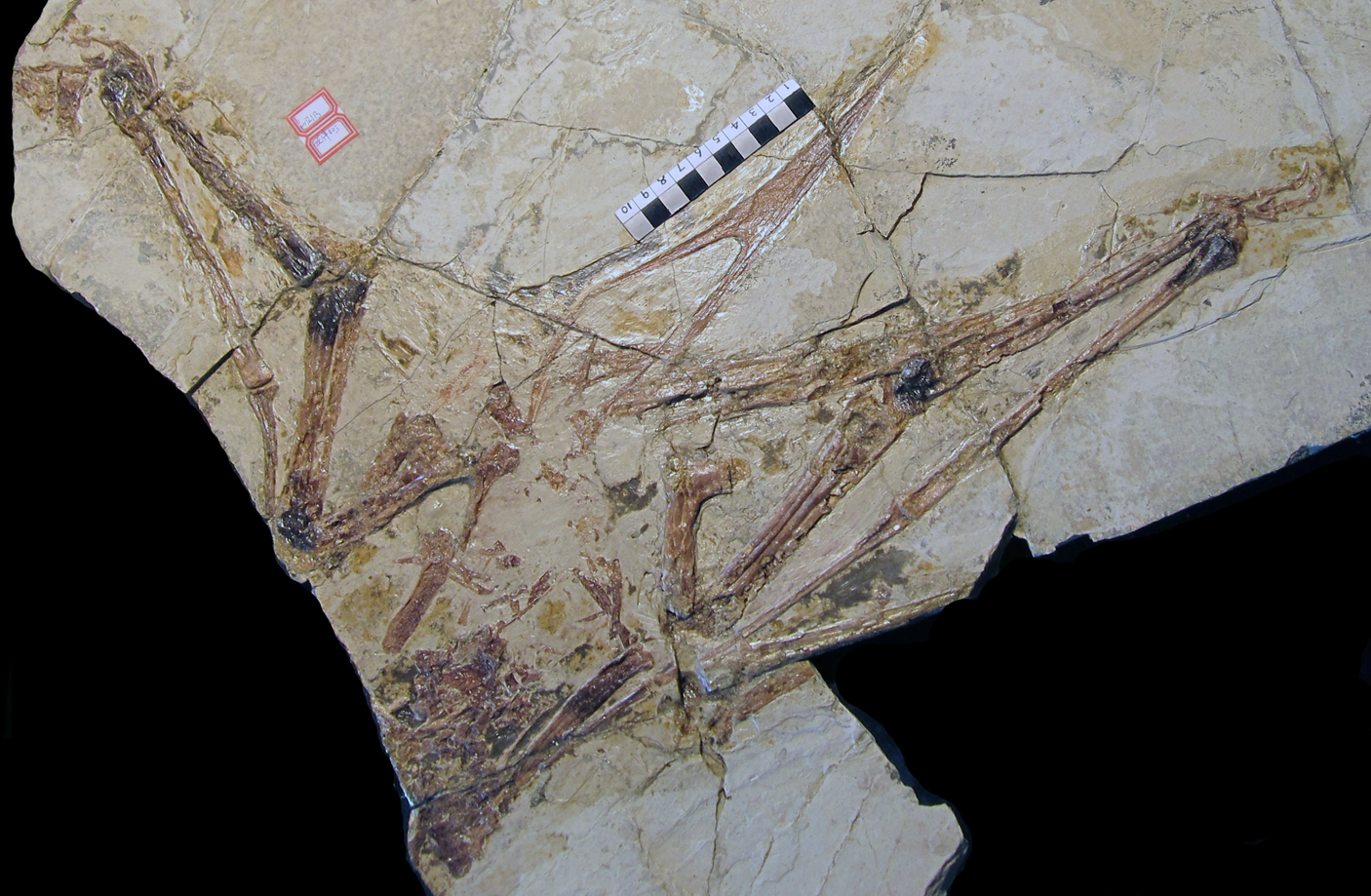

Supplement: S1 Fig — An incomplete skeleton includes skull, lower jaws, forelimbs and partial hindlimbs, collected from the Jiufotang Formation of Shangheshou, Chaoyang, western Liaoning. Scale bar = 10 mm. (TIF) [file pone.0185486.s002.tif]

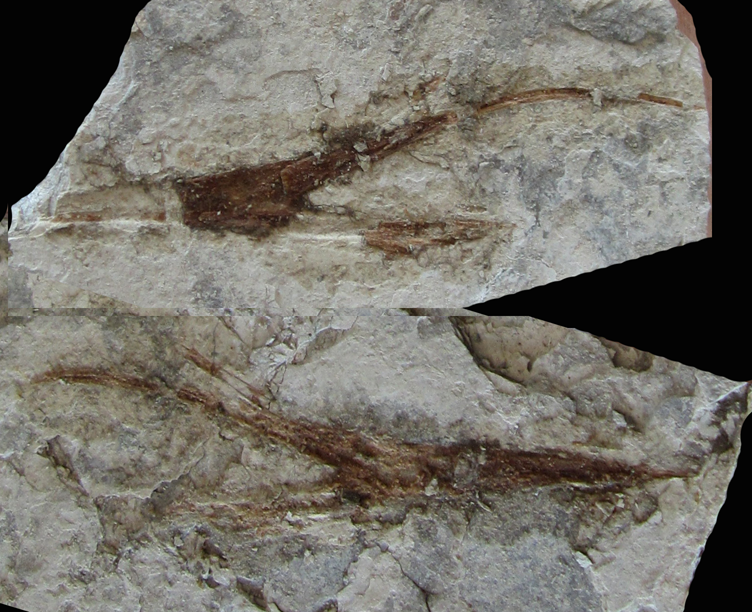

Supplement: S2 Fig — (TIF) [file pone.0185486.s003.tif]

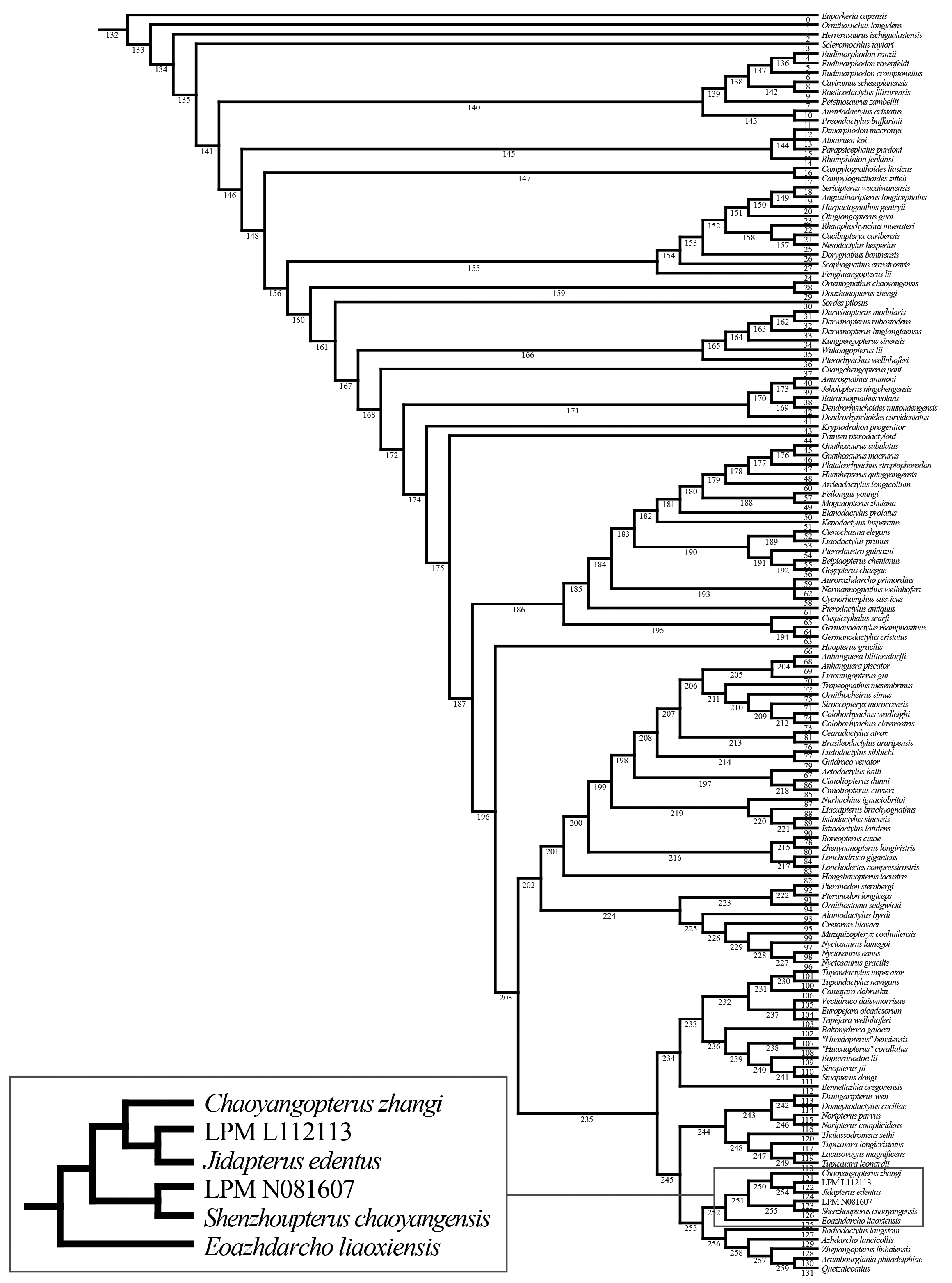

Supplement: S3 Fig — TNT node numbers are depicted below the branches that subtend the nodes. Branch lengths and support measures are listed in S1 Table. (TIF) [file pone.0185486.s004.tif]
